# The Plant Actin Cytoskeleton Responds to Signals from Microbe-Associated Molecular Patterns

**DOI:** 10.1371/journal.ppat.1003290

**Published:** 2013-04-04

**Authors:** Jessica L. Henty-Ridilla, Masaki Shimono, Jiejie Li, Jeff H. Chang, Brad Day, Christopher J. Staiger

**Affiliations:** 1 Department of Biological Sciences, Purdue University, West Lafayette, Indiana, United States of America; 2 Department of Plant Pathology, Michigan State University, East Lansing, Michigan, United States of America; 3 Department of Botany and Plant Pathology, and Molecular and Cellular Biology Program and Center for Genome Research and Biocomputing, Oregon State University, Corvallis, Oregon, United States of America; 4 Bindley Bioscience Center, Discovery Park, Purdue University, West Lafayette, Indiana, United States of America; Chinese Academy of Sciences, China

## Abstract

Plants are constantly exposed to a large and diverse array of microbes; however, most plants are immune to the majority of potential invaders and susceptible to only a small subset of pathogens. The cytoskeleton comprises a dynamic intracellular framework that responds rapidly to biotic stresses and supports numerous fundamental cellular processes including vesicle trafficking, endocytosis and the spatial distribution of organelles and protein complexes. For years, the actin cytoskeleton has been assumed to play a role in plant innate immunity against fungi and oomycetes, based largely on static images and pharmacological studies. To date, however, there is little evidence that the host-cell actin cytoskeleton participates in responses to phytopathogenic bacteria. Here, we quantified the spatiotemporal changes in host-cell cytoskeletal architecture during the immune response to pathogenic and non-pathogenic strains of *Pseudomonas syringae* pv. *tomato* DC3000. Two distinct changes to host cytoskeletal arrays were observed that correspond to distinct phases of plant-bacterial interactions *i.e.* the perception of microbe-associated molecular patterns (MAMPs) during pattern-triggered immunity (PTI) and perturbations by effector proteins during effector-triggered susceptibility (ETS). We demonstrate that an immediate increase in actin filament abundance is a conserved and novel component of PTI. Notably, treatment of leaves with a MAMP peptide mimic was sufficient to elicit a rapid change in actin organization in epidermal cells, and this actin response required the host-cell MAMP receptor kinase complex, including FLS2, BAK1 and BIK1. Finally, we found that actin polymerization is necessary for the increase in actin filament density and that blocking this increase with the actin-disrupting drug latrunculin B leads to enhanced susceptibility of host plants to pathogenic and non-pathogenic bacteria.

## Author Summary

The cytoskeleton is a dynamic platform for sensing and responding to a diverse array of biotic and abiotic stresses. The nature and timing of the changes in actin organization range from excessive bundling, to massive depolymerization, to new filament assembly, depending on the particular signal and the responding cell type. Here, we use the *Arabidopsis*–*Pseudomonas* pathosystem to dissect pathogen-derived cues that elicit changes in the plant host-cell cytoskeleton. Overall, we provide the first evidence that the actin cytoskeleton rearranges in response to a phytopathogenic bacterium and we quantified the temporal response of epidermal cells to *Pseudomonas syringae* pv. *tomato* DC3000 strains and susceptible *Arabidopsis* mutants, using a robust set of tools for measuring changes in actin organization. An immediate but transient increase in actin filament abundance was associated with pattern-triggered immunity. This response could be mimicked with microbe-associated molecular pattern peptide treatments. Second, we observed a late increase in actin filament bundling that appears to be part of effector-triggered susceptibility. We dissected the initial steps involved in the host-cell signaling pathway and demonstrated that FLS2, BAK1, and BIK1 were required for the actin response. Collectively, these findings demonstrate that rapid changes in host-cell cytoskeleton organization occur in response to receptor-mediated signaling during plant innate immunity.

## Introduction

Actin filament arrays in plant cells undergo constant remodeling and can respond rapidly to a diverse array of extracellular stimuli. Even in unstimulated epidermal cells, architectural rearrangements occur within seconds due to myosin-dependent translocation, remarkably fast filament assembly, and the destruction of filaments by prolific severing activity [1], [2]. This incessant remodeling of the actin cytoskeleton expends an enormous amount of energy, yet the physiological relevance of this is poorly understood. The actin cytoskeleton is a major signaling target and changes dramatically in response to numerous abiotic and biotic stimuli; the responses however are quite varied, ranging from filament bundling, to massive actin depolymerization, to assembly of new filament arrays [2]–[4]. For example, cells that are gently prodded with glass or tungsten needles generate extensively bundled filament arrays directly under the site of mechanical stimulation; yet, once the stimulus is removed the bundling is abrogated [5]. This is thought to mimic the efforts of fungi and oomycetes to gain entry into plant cells and, as such, it has been commonly assumed that attempted or actual penetration is responsible for eliciting changes in the host-cell actin cytoskeleton, rather than activation of host-cell defense signaling following the recognition of ‘non-self’. Actin filament arrays undergo a markedly different response upon recognition of ‘self’ pollen grains by a flower's stigma. Poppy pollen, for example, initiates a self-incompatibility (SI) response, resulting in massive depolymerization of actin filaments within minutes of stimulus perception, effectively inhibiting pollen tube growth and blocking fertilization [6]. In contrast with this signal-mediated destruction of actin filaments, the interaction between mutualistic bacteria and plant cells generally results in the development of bright phalloidin-decorated spots in host cells [7] – suggestive of actin polymerization. Other changes to actin during mutualistic interactions have been described, including filament reorientation from longitudinal to transverse arrays and increased numbers of actin bundles at the tip of root hairs; these responses can be reproduced with the application of purified Nod-factors from mutualistic bacteria onto host plant cells [8], [9]. Furthermore, *nap1* mutant root hairs, which are incapable of proper nodule formation, lack the ability to elicit changes to the actin cytoskeleton when Nod-factors are applied because these mutants are deficient for ARP2/3-dependent actin polymerization [10], [11]. On the other hand, certain signals from pathogenic fungi, like the *Verticillium dahlia* (VD) toxin, can stimulate dose-dependent destruction and relocation of cortical actin filaments to the perinuclear region [12]. Thus, biotic and abiotic signaling cascades produce a myriad of responses that can lead to dramatically different outcomes for actin organization and dynamics.

Plants are constantly exposed to a large number of fungal and bacterial microbes, however, most plants are immune to the majority of potential invaders due to a multilayered defense system. The initiation of plant immunity relies on structural defenses (*i.e.* the presence of trichomes, the closing of stomata to prevent bacterial entry, and the thickness and composition of the cell wall and cuticle) and inducible measures to guard the plant from various microbes [4]. These inducible processes can be classified in two nodes of defense signaling: pattern-triggered immunity (PTI) and effector-triggered immunity (ETI). PTI is a broad based immunity initiated through the host-cell recognition of conserved structural components, known collectively as microbe-associated molecular patterns or MAMPs, by cell-surface receptors [4], [13], [14], [15]. The recognition of microbes by the receptor kinase, FLAGELLIN-SENSING 2 (FLS2), is one of the best-studied PTI pathways in *Arabidopsis*. Upon perception of bacterial flagellin or the synthetic 22-amino acid peptide flg22, FLS2 associates with another receptor-like kinase, BAK1 (BRASSINOSTEROID INSENSITIVE1-ASSOCIATED KINASE 1); this association releases the cytoplasmic kinase BIK1 (BOTRYTIS-INDUCED KINASE 1) to induce down-stream defense signaling [4], [16]–[19], and ultimately prevents infection by non-adapted pathogens [4], [13], [14].

Pathogens elicit PTI in their respective host plants, and thus necessarily secrete or translocate various toxins and inject effector proteins into host cells to subvert PTI [14], [15]. For example, Gram-negative bacteria use a specialized type III secretion system (T3SS) to translocate collections of type III effector (T3E) proteins directly into host cells resulting in effector-triggered susceptibility (ETS) of the host plant [4], [13], [14]. The second node of immunity, ETI, relies on host-cell recognition and response to pathogen-specific effector proteins or their activities, to mount a defense response that is more rapid and more pronounced than PTI [4], [13], [14].

The plant actin cytoskeleton has been implicated in the generation and maintenance of many aspects of PTI. Major hallmarks of PTI in plant cells include endocytic uptake of receptors, changes to cytoplasmic streaming, activation of defense genes via mitogen-activated protein kinase (MAPK) signaling, recruitment of NADPH oxidase to the plasma membrane, an oxidative burst, directed trafficking of Golgi and endoplasmic reticulum to the site of attack, and callose deposition [3], [20]–[22]. The importance of an intact actin cytoskeleton for each of these responses has been demonstrated with pharmacological studies. The actin cytoskeleton is further assumed to play a central role in plant defense against microbes based on static images that show actin filament bundles impinging upon sites of both compatible and incompatible fungal or oomycete attack [3], [20], [21], [23]. Finally, disrupting the plant actin cytoskeleton with cytochalasin E treatment or by overexpressing actin-binding proteins allows penetration into plant cells and tissues by incompatible fungi and oomycetes [24]–[26].

Despite the growing body of evidence that suggests the involvement of the actin cytoskeleton in either PTI or ETI, no direct evidence linking specific aspects of either layer of immune signaling have been reported. However, a recent report shows that a T3E protein from *Pseudomonas syringae*, HopZ1a, targets the microtubule cytoskeleton to circumvent PTI [27]. Similarly, one report describes actin filament stabilization through monoubiquitination of actin during infection by either pathogenic or mutualistic bacteria, but not in response to stress or viral infection [28]. We hypothesize that the continuous rearrangements of the actin cytoskeleton in *Arabidopsis* epidermal cells represent a surveillance mechanism to external threats [1]; however, it is still unclear whether there are direct links between actin and PTI- or effector-mediated signal transduction cascades or which actin-binding proteins are involved. Significantly, changes in the expression of Actin Depolymerizing Factor (ADF) enhance non-host susceptibility in both fungal and bacterial pathosystems, but these changes do not alter the focal accumulation of profilin at pathogen-invasion sites or expression of defense genes [26]. Notably, the localization of *Arabidopsis* ADF4 to the nucleus correlates with the reduced expression of the hallmark PTI-defense gene, *FRK1*, which implicates the actin cytoskeleton in the early onset of PTI [29]. Additionally, this *adf4* knockout mutant fails to activate ETI in response to *P. syringae* expressing the cognate bacterial effector gene *AvrPphB* [30]. Collectively, these results suggest that actin organization and dynamics are strictly regulated in both PTI and ETI. To date, the timing and nature of actin-based responses in host cells during bacterial pathogen attack have not been described.

Using a combination of bacterial mutants and advanced imaging of actin cytoskeleton organization in epidermal cells from *Arabidopsis*, we analyzed the host-cell response to the bacterial phytopathogen *P. syringae* pv. *tomato* DC3000. We quantified the nature of specific changes in actin array architecture over a time-course of infection with both pathogenic and non-pathogenic strains of bacteria. A transient increase in the density of actin filament arrays in the cortex of epidermal cells was identified, and we demonstrate that this change did not require either the T3SS or effector proteins. Moreover, we found that infiltration of leaves with MAMPs was sufficient to elicit an increase in actin filament density. Using reverse genetics, we have also begun to dissect the plant signaling pathways required to elicit actin rearrangement during PTI. Notably, we found that FLS2, BAK1 and BIK1 were required for the increase in actin filament density. When actin polymerization was blocked by treatment with latrunculin B, the increase in actin filament density did not occur and plants were more susceptible to infection with pathogenic and non-pathogenic bacteria. These data implicate the transient increase in cytoskeletal array density as a contributing factor during PTI and identify parts of the signal transduction machinery necessary for this response.

## Results

### *Arabidopsis* Cotyledons Support the Growth of the Phytopathogenic Bacterium *P. syringae* pv. *tomato* DC3000

In this study, we focused on cytoskeletal responses in the *Arabidopsis–Pseudomonas* pathosystem and used seedlings expressing a well-characterized actin reporter, GFP-fABD2. Dip-inoculated cotyledons from wild-type Col-0 and transgenic plants expressing GFP-fABD2, a fusion protein between green fluorescent protein and the second actin-binding domain of *Arabidopsis* FIMBRIN1 [1], exhibited necrotic lesions when inoculated with pathogenic *P. syringae* pv. *tomato* DC3000 (hereafter referred to as DC3000; Figure S1A & C), whereas cotyledons inoculated with the non-pathogenic T3SS-deficient mutant *hrpH* did not (Figure S1B & D). Furthermore, cotyledons (Figure S1E) and rosette leaves (Figure S1F) inoculated with DC3000 had a higher bacterial load than those inoculated with *hrpH* at 4 days after inoculation. These results confirm that DC3000 can proliferate and cause disease symptoms on seedling cotyledons expressing GFP-fABD2, and that bacterial growth is not significantly different when bacteria are infected in cotyledons or rosette leaves.

### Changes to Actin Filament Architecture Occur during the Plant Immune Response to Bacterial Pathogens

**Figure 1:**
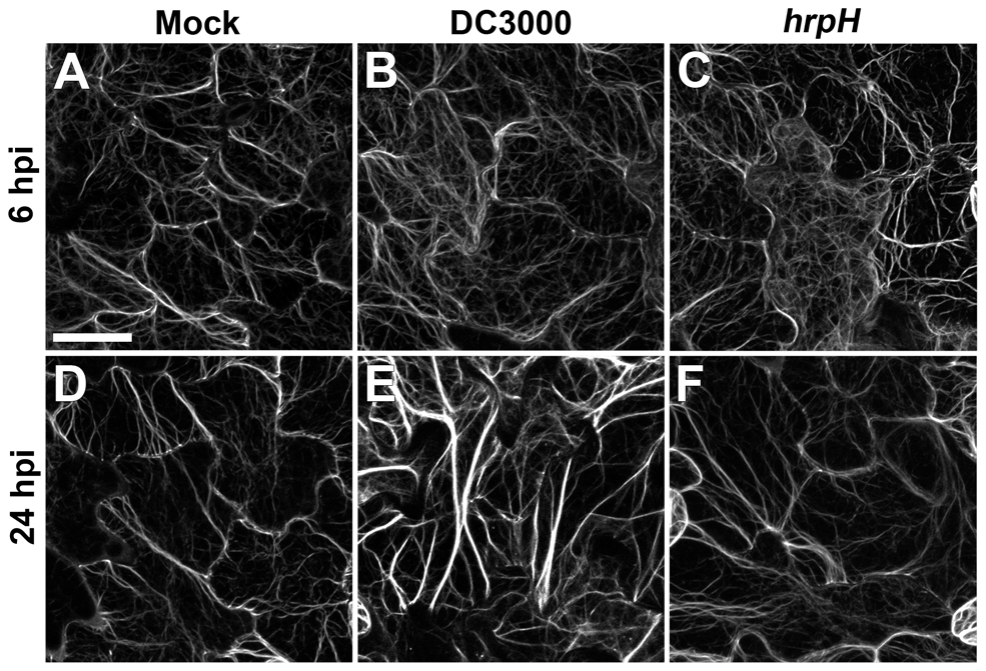
*Arabidopsis* seedlings exhibit changes in actin organization in response to *P. syringae* pv. *tomato* DC3000. Representative images of epidermal pavement cells from *A. thaliana* cotyledons expressing the actin reporter GFP-fABD2. Images show an apparent increase in filament abundance at 6 hpi when treated with *Pseudomonas syringae* pv. *tomato* DC3000 (**B**) or the T3SS-deficient mutant *hrpH* (**C**), compared to mock treated (**A**). At 24 hpi, an increase in the extent of filament bundling is observed following inoculation with DC3000 (**E**), compared to *hrpH* (**F**) and mock (**D**). Epidermal cells from 10 d-old light-grown seedlings were imaged with spinning disk confocal microscopy (SDCM), and micrographs shown are z-series projections compiled from 24 optical sections. Bar = 15 µm.

To study the response of host-cell cytoskeleton during bacterial infection, we imaged actin filament arrays in cotyledons with spinning disk confocal microscopy (SDCM) at various time-points after dip-inoculation with DC3000 and *hrpH* (Figure 1). Epidermal pavement cells from *Arabidopsis* cotyledons, display two populations of actin filaments in the cortical cytoplasm—dynamic, faint structures that resemble single actin filaments; and, thick, bright actin filament bundles (Figure 1A & D). At 6 hours post inoculation (hpi), we observed an increase in the abundance of actin filaments in the cortical array of epidermal cells inoculated with DC3000 (Figure 1B) or *hrpH* (Figure 1C), compared to mock-treated material (Figure 1A). At 24 hpi, we noticed little difference between the mock control (Figure 1D) and *hrpH* inoculation (Figure 1F); however, obvious actin filament bundling occurred following DC3000 treatment (Figure 1E).

**Figure 2:**
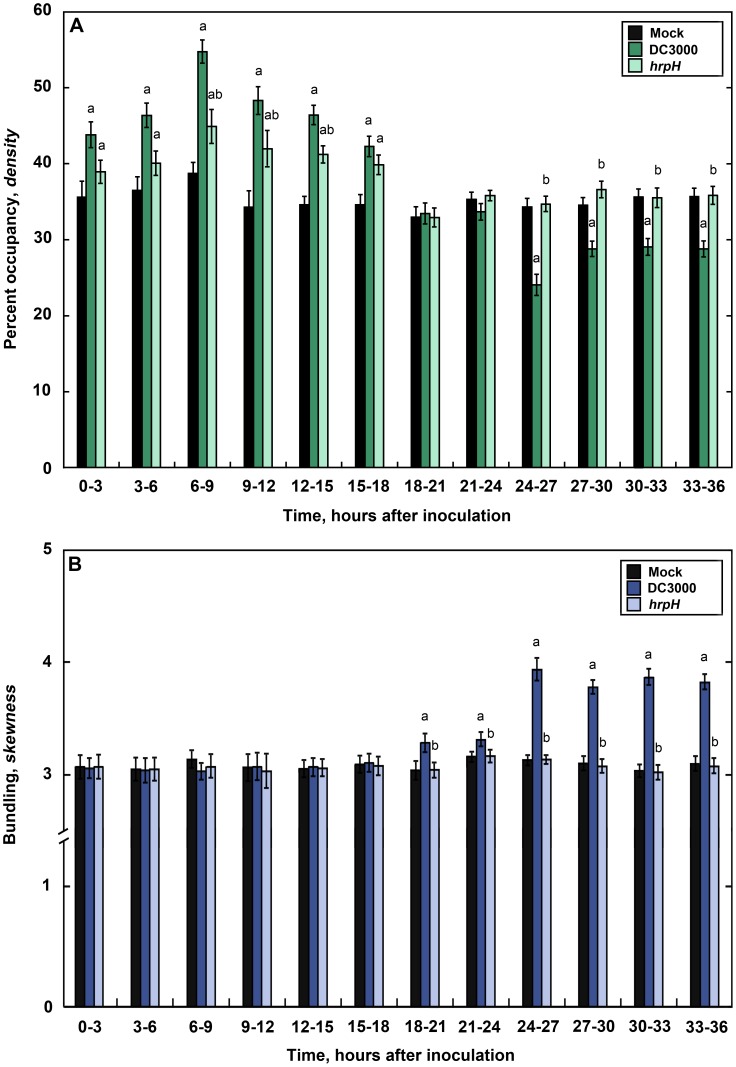
Changes in actin filament organization in response to inoculation with pathogenic and non-pathogenic *P. syringae*. Actin architecture parameters for percent occupancy (**A**) and extent of filament bundling (**B**) were measured over time in response to *P. syringae* inoculation. Percent occupancy increases transiently in epidermal cells of DC3000-inoculated and *hrpH*-inoculated cotyledons at 0–18 hpi. Filament *density* returns to mock-treated levels at 18–21 hpi and then significantly decreases at 24–36 hpi in epidermal cells from DC3000-inoculated cotyledons. Seedlings treated with *hrpH*, however, do not show the same reduction in percent occupancy from 24 hpi onwards. The presence of actin filament bundles in DC3000-inoculated seedlings is significantly enhanced at 18–36 hpi. In contrast, *hrpH*-inoculated cotyledons show no change in filament bundling at any timepoint measured. Images were collected as described for Figure 1. Values given are means ± SE (*n* = 105–150 images per treatment, per timepoint, from *n* = 3 biological repeats). Significant differences by ANOVA, with Tukey HSD post-hoc analysis, are represented as follows: a, *P*≤0.05 between mock and treatment; b, *P*≤0.05 between DC3000 and treatment.

To further investigate the timing and nature of actin responses following DC3000 inoculation, we used a set of previously validated tools for measuring actin array organization [31]–[33]. We quantified and statistically compared maximum intensity projections generated from fields of *Arabidopsis* epidermal cells for changes in the extent of actin filament bundling (*skewness*) and percent occupancy (*density*) following microbial infection. The bundling parameter is based on the assumption that a population of individual actin filaments will have a Gaussian distribution of pixel intensities, which becomes *skewed* in favor of brighter pixels, when the array of actin filaments becomes more bundled [31]. The *density* metric is calculated as the percent occupancy of GFP-fABD2-containing pixels in each micrograph [31]. For these analyses, we performed a time-course from 0 to 36 h after DC3000 infection by sampling at 3-h intervals (Figure 2). We observed a transient increase in the abundance of actin filaments in host cells at 0–15 hpi following DC3000 treatment (Figure 2A) and this occurred as early as 15–30 min after inoculation (Figure S2). Actin filament abundance was elevated by as much as 16%, with a peak at 6–9 hpi, and then significantly decreased from 24–36 hpi onward (Figure 2A). We also detected significantly enhanced filament bundling at 18–36 hpi, with the most prominent bundling at 24–27 hpi (Figure 2B). Mock-treated seedlings had no significant changes in actin architecture compared to untreated seedlings (Figure S3). These results revealed two distinct and statistically significant changes in actin filament organization following infection with virulent pathogen, *i.e.* an early and transient increase in actin filament density as well as a late increase in the extent of actin filament bundling. These observations are consistent with the immediate perception of DC3000 and response of the plant immune system followed by a subsequent suppression of PTI by the pathogen.

### Actin Filament Density Increases in Response to Diverse PTI-eliciting Microbes

**Figure 3:**
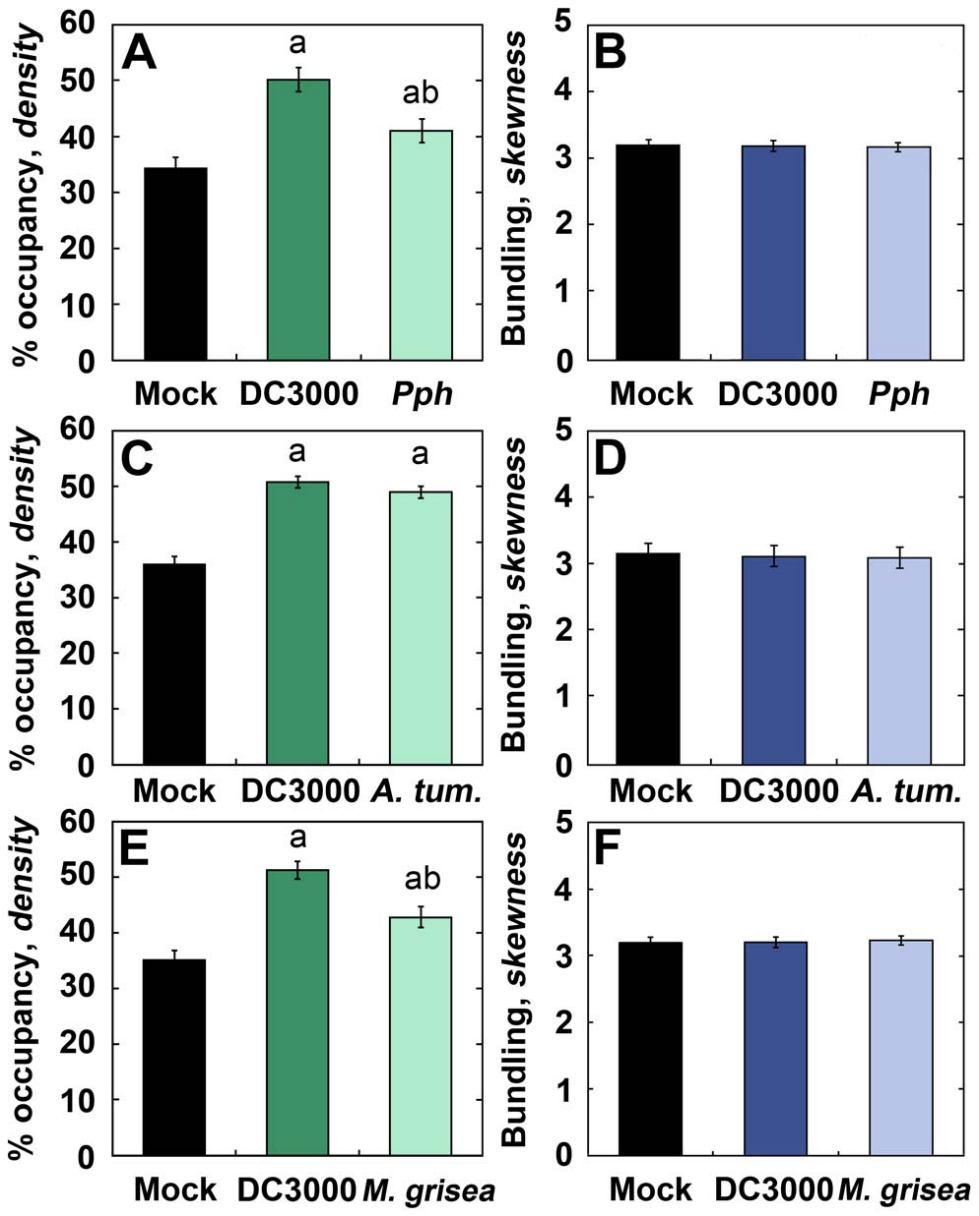
Diverse PTI-inducing microbes elicit an increase in actin filament density. Actin architecture analysis of cotyledons inoculated with *P. syringae* pv. *phaseolicola* (*Pph*; [**A**]), *Agrobacterium tumefaciens GV3101* (*A. tum.*; [**C**]), or *Magnaporthe grisea* (**E**) display a significant increase in actin filament density at 6–9 hpi. In contrast, bundling was not significantly different from mock-treated controls following *Pph* (**B**), *A. tumefaciens* (**D**), or *M. grisea* (**F**) inoculations. Values given are means ± SE (*n* = 150 images per treatment, from *n* = 3 biological repeats). Significant differences by ANOVA, with Tukey HSD post-hoc analysis, are represented as follows: a, *P*≤0.05 between mock and treatment; b, *P*≤0.05 between DC3000 and treatment.

If the rapid and transient increase in actin filament density in epidermal cells exposed to DC3000 is part of the PTI response, then we predict that the same cytoskeletal change will occur with various phytopathogens that are not adapted to *Arabidopsis*. To test this, we quantified actin array architecture in cotyledons at 6–9 h following infection with several non-adapted pathogens that trigger PTI in *Arabidopsis*, including the bean pathovar *P. syringae* pv. *phaseolicola* (*Pph*); *Agrobacterium tumefaciens*; and the rice-blast fungus *Magnaporthe grisea* (Figure 3). Following *Pph* inoculation, we observed a significant increase in actin filament density (Figure 3A), but no change in the extent of filament bundling (Figure 3B). Additionally, we observed increased actin filament density with *A. tumefaciens* (Figure 3C) and *M. grisea* (Figure 3E) treatments, but no change in bundling (Figure 3D & F). In summary, the density or abundance of actin filaments is elevated in cotyledons following treatment with various bacterial and fungal microbes and likely represents a broad-based PTI response.

### Increased Actin Filament Density in Cotyledons Is Conserved Following Inoculation with *Pseudomonas* Mutants

**Figure 4:**
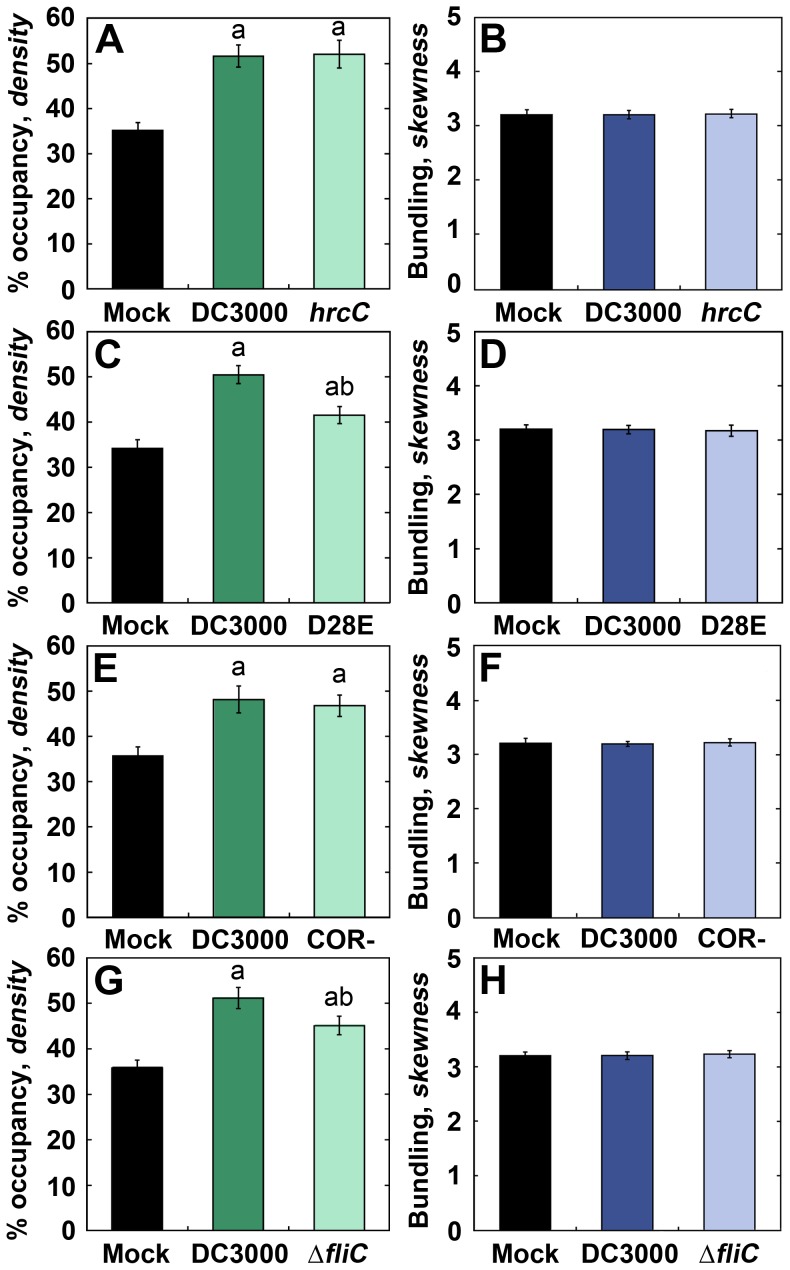
Analysis of *P. syringae* mutants implicate a role for PTI in the filament density change. Actin architecture analysis of epidermal cells treated with the T3SS-deficient mutant *hrcC* (**A**), the effectorless mutant D28E (**C**), the coronatine-deficient mutant COR- (**E**), and the flagellin deletion mutant *ΔfliC* (**G**) exhibit significantly enhanced actin filament density compared to mock-treated controls at 6–9 hpi. However, bundling analysis of the same images used in (**A, C, E, & G**) shows no significant change from mock-treated following *hrcC* (**B**), D28E (**D**), COR- (**F**), and *ΔfliC* (**H**) inoculation. Images were collected at 6–9 hpi. Values given are means ± SE (*n* = 150 images per treatment, from *n* = 3 biological repeats). Significant differences by ANOVA, with Tukey HSD post-hoc analysis, are represented as follows: a, *P*≤0.05 between mock and treatment; b, *P*≤0.05 between DC3000 and treatment.

Since the increase in actin filament abundance was observed in host cells in response to both adapted and non-adapted microbes (Figure 3), we predict that the increased actin filament density occurs independent of the T3SS or translocated effector proteins. Therefore, we used genetic mutants to dissect the molecular nature of *P. syringae*'s ability to elicit the host-actin response. First, we quantified actin filament architecture in cotyledons treated with the T3SS-deficient mutant *hrpH* over a full time-course and observed a significant increase in percent occupancy from 0–15 hpi following inoculation (Figure 2A). Actin filament density peaked at 6–9 hpi, similar to DC3000; however, no decrease in density at 24–36 hpi was observed (Figure 2A). In contrast to DC3000, no increase in filament bundling was observed at any time-point following *hrpH* inoculation (Figure 2B). The similar responses to DC3000 and *hrpH* inoculations at 0–15 hpi further support the argument that the transient increase in actin filament density is PTI-based. Moreover, because *hrpH* does not induce bundling in epidermal cells whereas DC3000 does, it is likely that bundling is associated with effector-triggered susceptibility (ETS). To further dissect whether the actin organization changes were part of a general response to bacteria or can also be influenced during ETI, we quantified the actin filament architecture over a full time-course in *Arabidopsis* cotyledons following inoculation with *P. syringae* DC3000 expressing the YopT homolog, AvrPphB ([34]–[36]; Figure S4). Similar increases in actin filament density were observed with DC3000 and DC3000 expressing AvrPphB inoculations at 0–15 hpi (Figure S4A). In contrast, filament bundling was not as pronounced with DC3000 expressing AvrPphB inoculation compared to DC3000 (Figure S4B) and the filament density did not decline at 24–36 hpi, suggesting that these later changes in filament array architecture are part of a gene-for-gene response or ETI. Finally, we quantified cortical actin architecture in cotyledons treated with another T3SS-deficient mutant, *hrcC* [37], and a D28E mutant that expresses the T3SS but lacks most T3E genes ([38]; Figure 4). Following *hrcC* or D28E inoculation, we observed a significant increase in percent occupancy similar to treatments with *hrpH* and DC3000 (Figure 4A & C), but no change in filament bundling at 6–9 hpi (Figure 4B & D). *Sensu stricto*, these results demonstrate that the early increase in actin filament density is associated with PTI.

As an alternative or in addition to T3SS activity, *P. syringae* might alter the host-cell actin cytoskeleton through the secretion of pathotype-specific toxins, such as coronatine, a jasmonate mimic synthesized by DC3000 [39]. Following inoculation of cotyledons with the coronatine-deficient mutant, COR- [37], we observed a significant increase in filament density in host cells (Figure 4E) but no change in filament bundling (Figure 4F). This confirms that less virulent bacteria still elicit an increase in actin filament density and that coronatine is not necessary for DC3000 to elicit changes in the cytoskeleton of epidermal cells.

To further examine the mechanism of increased actin filament density during PTI, we inoculated cotyledons with a mutant of *P. syringae* that has the flagellin gene deleted (Δ*fliC*; [40]) and quantified changes in actin organization. We observed a significant increase in filament density (Figure 4G) and no change in bundling (Figure 4H). Although this increase in filament abundance was significantly less than the increase associated with DC3000 treatment, flagellin is not strictly necessary to elicit the increase in actin filament density. These results imply that the perception of other MAMPs can also lead to changes in actin organization.

### Treatment with flg22 Peptide Mimics the Increase in Filament Density Induced by Microbes

**Figure 5:**
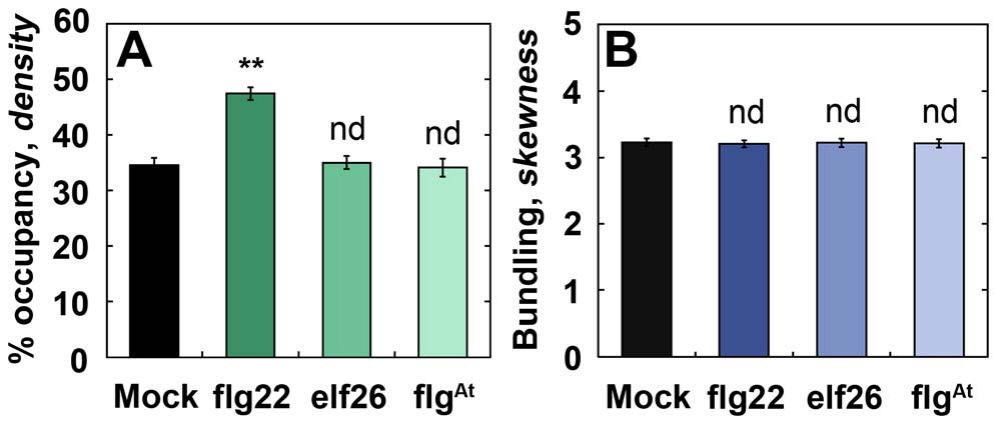
Treatment with flg22 peptide is sufficient to increase filament density. Twenty-four d-old plants that were hand-infiltrated with 1 µM flg22 peptide show significantly enhanced filament *density* (**A**), whereas the same concentration of either elf26 or *A. tumefaciens* flg (flg^At^) peptide exhibit no change compared to mock controls. Bundling analysis (**B**) of the same images used in (**A**) shows no significant change for any MAMP peptide treatment. Epidermal pavement cells from rosette leaves were imaged by SDCM at 0–3 hpi and 24 optical sections were combined into a z-series projection. Values given are means ± SE (*n* = 150 images per treatment, from *n* = 3 biological repeats). Asterisks represent significant differences by ANOVA.

To investigate whether MAMPs are sufficient to elicit a change in actin organization, we challenged plants with synthetic MAMP peptides, as well as with the fungal glucosamine polymer, chitin, and monitored changes in actin architecture. We used flg22, the N-terminal twenty-two amino acids from *Pseudomonas* flagellin [41], [42]; elf26, a twenty-six amino acid peptide from bacterial EF-Tu [43]; and flg^At^, the amino terminal sequence from *Agrobacterium* flagellin which does not elicit a response in *Arabidopsis* [42]. These peptides were introduced at various concentrations into mature leaves and the actin responses at 0–3 h after infiltration were quantified (Figure 5 & S5). With flg22, we detected a rapid and dose-dependent increase in actin filament density (Figure 5A & S5A); however, no change in the extent of filament bundling was detected at any concentration tested (Figure 5B & S5B). We detected similar changes to actin filament arrays in plants infiltrated with chitin (Figure S5G & H). In contrast, infiltration with elf26 or flg^At^ was indistinguishable from mock at all concentrations tested (Figure 5 & S5C–F) even though, all three MAMP peptides were able to stimulate a PTI-based defense response as demonstrated by activation of *FRK1* transcripts (Figure S6). In conclusion, treatments with flg22 or chitin are sufficient to induce rapid changes in actin filament organization.

### Recognition of MAMPs by an *Arabidopsis* Receptor Complex Is Required for Increased Actin Filament Density

**Figure 6:**
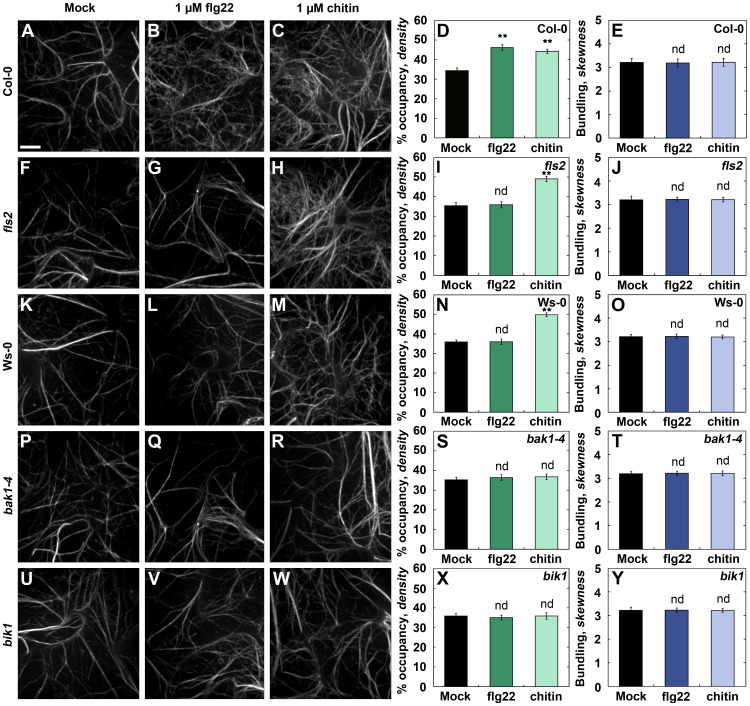
*Arabidopsis* knock-out mutants define the early signaling steps required for increased actin filament density. Actin architecture analysis of epidermal cells was performed on 24 d-old *Arabidopsis* mutants that were hand-infiltrated with 1 µM flg22 peptide or chitin oligomers. Treatment with 1 µM flg22 (**B & D**) or 1 µM chitin (**C & D**) is sufficient to increase filament abundance in wild-type Columbia-0 plants, whereas mock-treatment does not elicit a change to actin architecture (**A & D**). Actin architecture analysis of mock (**F & I**)- or flg22 (**G & I**)-treated epidermal cells from *flagellin-sensing 2* (*fls2*) knock-out mutants did not elicit a change in filament abundance, however, chitin-treatment (**H & I**) did. Similar to *fls2*, actin architecture in wild-type Ws-0 plants was not changed with mock (**K & N**)- or flg22-treatment (**L & N**), however treatment with chitin (**M & N**) was sufficient to increase filament density. Finally, actin architecture analysis of epidermal cells from *brassinosteroid insensitive1-associated receptor like kinase1* (*bak1-4*) or *botrytis induced kinase1* (*bik1*) homozygous mutant plants did not exhibit significantly different changes in filament density from mock (for *bak1-4*: [**P & S**]; for *bik1*: [**U & X**]) with either flg22 (for *bak1-4*: [**Q & S**]; for *bik1*: [**V & X**]) or chitin (for *bak1-4*: [**R & S**]; for *bik1*: [**W & X**]) treatments. No significant changes to bundling compared with mock respective controls were observed (**E, J, O, T & Y**). Epidermal pavement cells from rosette leaves were imaged by SDCM at 0–3 hpi and 24 optical sections were combined into a z-series projection. Values given are means ± SE (*n* = 150 images per treatment, from *n* = 3 biological repeats). Asterisks represent significant differences by ANOVA.

To examine the role of early cellular signaling pathways during PTI, we performed actin architecture analysis on several *Arabidopsis* knockout mutants, including a susceptible *Arabidopsis* ecotype, infiltrated with either 1 µM flg22 or 1 µM chitin (Figure 6). Wild-type Col-0 plants showed significantly enhanced filament abundance following treatment with flg22 (Figure 6B & D) or chitin (Figure 6C & D), compared with mock treatment (Figure 6A & D). The homozygous flagellin receptor mutant, *flagellin sensing-2* (*fls2*), in the Col-0 background, as well as the *Fls2*-deficient ecotype Wassilewskija-0 (Ws-0), both lacked a significant increase in filament abundance following treatment with flg22 (Figure 6G & I and Figure 6L & N, respectively). Whereas with chitin treatment, actin filament abundance was significantly increased following treatment of epidermal pavement cells in either *fls2* (Figure 6H & I) or Ws-0 (Figure 6M & N) plants. To further test which signaling pathways may involve actin, we performed actin filament density analysis on two additional DC3000-susceptible *Arabidopsis* knockout mutants, *brassinosteroid insensitive1-associated kinase1* (*bak1-4*) and *botrytis-induced kinase 1* (*bik1*), which are both known to associate with the FLS2 receptor [17], [18]. Neither *bak1-4* (Figure 6P–T) nor *bik1* (Figure 6U–Y) homozygous mutant plants showed significant changes from mock following treatment with flg22 or chitin. Additionally, no significant changes to filament bundling were observed following treatment with either flg22 or chitin for any of the plant lines tested. Since flg22 peptide and chitin are sufficient to elicit an increase in actin filament abundance, we tested whether *Pseudomonas* bacteria could elicit a similar response in *Arabidopsis* lines with altered defense signaling. Wild-type Col-0, the Ws-0 ecotype, and the *fls2* knockout mutant were dip-inoculated, individually, with multiple *Pseudomonas* mutants and changes to the host cytoskeleton at 6–9 hpi were investigated (Figure S7). Notably, the actin response was reduced in both the *fls2* mutant (Figure S7C) and the Ws-0 ecotype (Figure S7E), but not completely ameliorated, indicating that some changes to the actin cytoskeleton are independent of FLS2. However no significant changes to the extent of actin filament bundling were observed with any *Pseudomonas* strain or host-plant at this time-point (Figure S7B, D & F).

### Disruption of Host-Actin Arrays Promotes *P. syringae* Virulence

**Figure 7:**
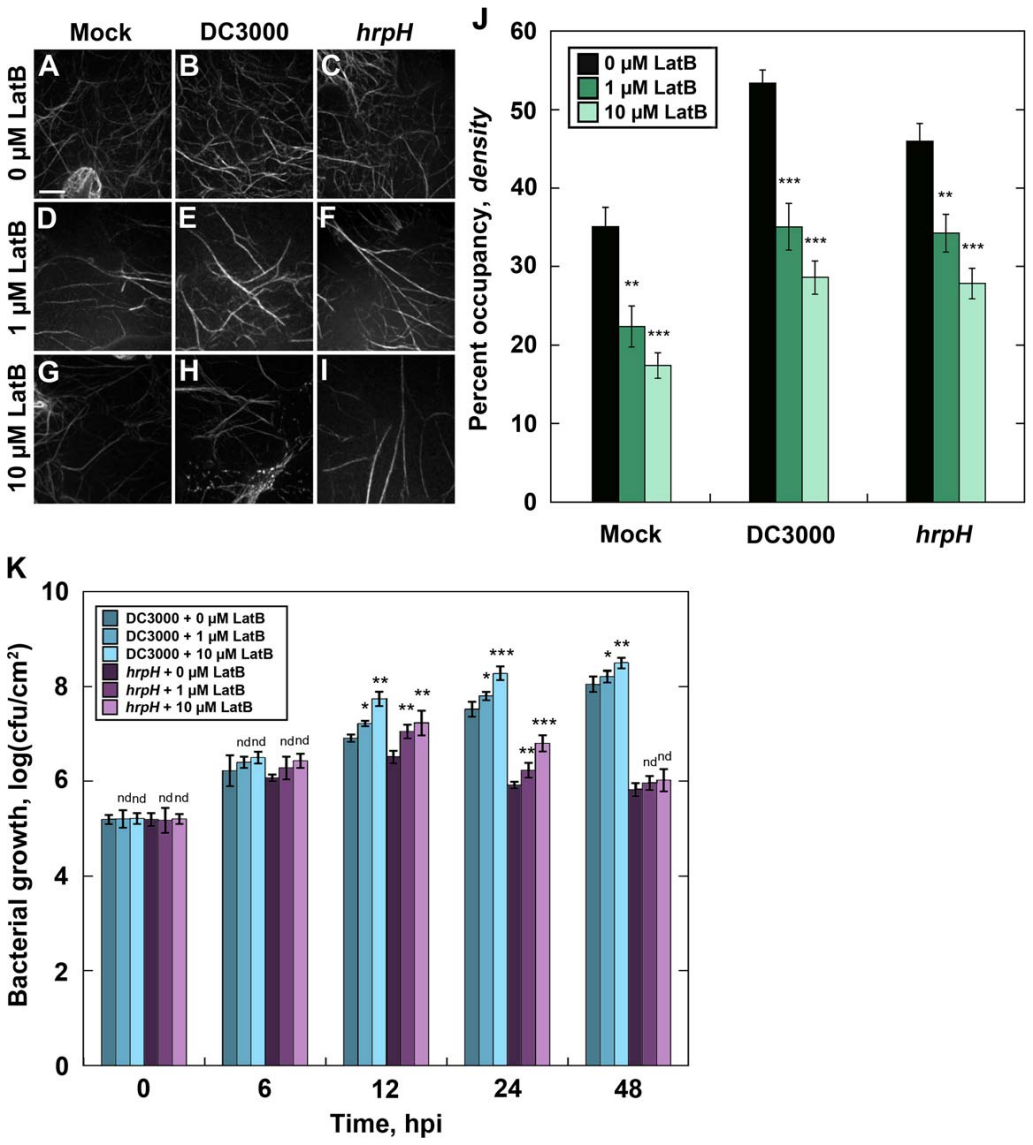
Disruption of the host-cell actin cytoskeleton promotes virulence. The consequences of disrupting the host-cell actin cytoskeleton with latrunculin B (LatB) were measured by microscopy and by quantifying the amount of bacterial growth on co-infiltrated leaves. Representative images of epidermal pavement cells from *Arabidopsis* show an apparent increase in filament abundance at 6 hpi when treated with DC3000 or *hrpH* in the absence of LatB (**A–C**). However, actin arrays appear less abundant in epidermal cells of plants following treatment with 1 µM (**D**) or 10 µM LatB (**G**) but not treated with bacteria (i.e. mock). Further, the apparent increase in actin filament abundance appears severely reduced following co-infiltration of LatB and DC3000 (**E & H**) or *hrpH* (**F & I**). Significant decreases in actin filament abundance were measured for plants co-infiltrated with DC3000 or *hrpH* at various concentrations of LatB (**J**). Disruptions to the host-cell actin cytoskeleton elicited a significant increase in bacterial growth following co-infiltration with LatB and DC3000 or *hrpH* (**K**). Epidermal pavement cells from rosette leaves of 24 d-old wild-type *Arabidopsis* plants were imaged by SDCM at 6–9 hpi and 24 optical sections were combined into a z-series projection. Values given are means ± SE (*n* = 30 images per treatment). LatB co-infiltration experiments were repeated 3 times and the mean results are presented ± SE. Asterisks represent significant differences by ANOVA. nd, no significant difference compared to 0 µM treatment; *, *P*≤0.05; **, *P*≤0.001; ***, *P*≤0.0001.

Latrunculin B (LatB) is a macrolide compound from marine sponges that inhibits actin polymerization by binding to monomeric actin and preventing its assembly onto filament ends [44]. To test whether actin polymerization is necessary for the increase in filament density during the initial response to phytopathogens, we co-infiltrated *Arabidopsis* leaves with various concentrations of LatB and DC3000 or *hrpH Pseudomonas* strains. At 6–9 hpi, actin filaments in epidermal pavement cells appeared to be markedly reduced following infiltration with LatB alone (Figure 7A, D & G) or LatB co-infiltrated with either DC3000 (Figure 7B, E & H) or *hrpH* (Figure 7C, F & I). As shown previously [1], short-term treatments with low doses of LatB primarily affected the dynamic actin filament arrays and individual filaments, whereas only modest effects on filament bundles were observed (Figure 7D–I). Using the metrics described earlier, we measured a significant decrease in actin filament density following LatB infiltration in mock-treated plants compared to the 0 µM control (Figure 7J). Further, we measured a significant and dose-dependent reduction in actin filament abundance in plants that were co-infiltrated with LatB and DC3000 or *hrpH* (Figure 7J). However, no significant changes to the extent of filament bundling were observed at this early stage of infection (P-value = 0.49, ANOVA; data not shown). This demonstrates that actin polymerization is necessary for the increase in filament density following infection with pathogenic and non-pathogenic bacteria during the PTI response.

If actin polymerization is an important component of PTI, then blocking this aspect of the host-cell response should enhance susceptibility to pathogens. To test this, we again co-infiltrated *Arabidopsis* plants with various concentrations of LatB and either DC3000 or *hrpH* and measured bacterial growth at 0, 6, 12, 24, and 48 h after treatment. As predicted, bacterial growth was significantly increased in plants co-infiltrated with DC3000 and 1 or 10 µM LatB compared to 0 µM LatB treatments (Figure 7K). We also measured a significant increase in bacterial growth following co-infiltration with the non-pathogenic strain, *hrpH*, and LatB (Figure 7K) indicating that the host-actin cytoskeleton and actin polymerization are necessary for innate immunity.

## Discussion

Using robust tools for measuring actin organization [31]–[33], we quantified temporal changes to actin arrays in *Arabidopsis* seedlings infected with the phytopathogenic bacterium *P. syringae* pv. *tomato* DC3000. Here we provide the first report that changes in plant actin array organization occur in response to pathogenic bacteria. We observed an early transient increase in the density of actin filament arrays that was corroborated by treatments with flg22 MAMP peptide, and chitin, as well as with several non-host adapted pathogens. We also demonstrated that several components of host-cell signaling (*i.e.* FLS2, BAK1, and BIK1) were necessary for this response, providing the first evidence for receptor-mediated signaling to the actin cytoskeleton during plant innate immunity. Finally, we used the pharmacological agent LatB to demonstrate that actin polymerization and an increase in filament abundance are necessary for innate immunity. Collectively, these observations demonstrate unambiguously that the rapid increase in actin filament abundance is a component of PTI, requiring recognition of a bacterial MAMP by an *Arabidopsis* MAMP-receptor complex. Secondly, we observed a decrease in actin filament abundance and a late increase in filament bundling that was only associated with pathogenic DC3000 in a T3SS-dependent manner. Therefore, we suggest that these changes are a consequence of the action of T3E proteins.

### Increased Actin Filament Density: A Fast Response to Perception of Microbes in Beneficial and Pathogenic Interactions

The increase in actin filament density observed in *Arabidopsis* cotyledons is associated with a rapid PTI response; it could be detected as early as 15–30 min after inoculation with DC3000. This response peaked at 6–9 hpi and was abrogated by 15 hpi. Further, this increase occurred in cotyledons inoculated with pathogenic and non-pathogenic *Pseudomonas* strains and mutants. It also occurred when seedlings were inoculated with *Agrobacterium tumefaciens*, and the non-host fungus *Magnaporthe grisea*, which indicates that the increase in actin filament density is a broad-based response to microbial perception. The increase in actin filament density is not the result of mechanical stimulation by the T3SS, as two T3SS-deficient mutants (*hrpH* and *hrcC*) still elicited the early cytoskeletal response. Although we observed significantly less actin filament percent occupancy following D28E inoculation compared to DC3000, the density of filaments was still significantly higher than the mock-treated control. Since each of the *P. syringae* mutants utilized herein is thought to contain the same suite of MAMPs (with the notable exception of *ΔfliC*), this significant reduction in filament abundance may imply a reduction in bacterial growth or indicate the action of T3E proteins in this response. The latter possibility seems unlikely as plants treated with *P. syringae* DC3000 expressing AvrPphB led to an similar actin response at early time-points and a reduced filament and bundling response at late time-points.

Changes to actin filament density, usually described as increases in polymerization [9] or an increase in phalloidin-stained spots [7], are also observed with mutualistic bacteria as well as with compatible and incompatible fungal and oomycete interactions [3], [20]–[23]. Despite the commonly accepted dogma that actin responses are caused by the mechanical stress of invading pathogens [5], [21]–[23], our data indicate that host-cell penetration by an invading pathogen is not necessary to elicit changes to the actin cytoskeleton. Here, we show that *Magnaporthe* spores, which do not attempt to penetrate *Arabidopsis* epidermal cells, still elicit an increase in actin filament abundance. Previously, a *Magnaporthe* mutant that is deficient for the ability to penetrate cells could also stimulate rearrangement of the host cytoskeleton in onion epidermal cells, leading the authors to speculate that this was due to sensing fungal cues other than the penetration peg [45]. Unlike fungi and oomycetes which use specialized structures to penetrate host cells, phytopathogenic bacteria reside in intercellular spaces; as a result, signals indicating the presence of these bacteria may affect multiple cells, rather than a single point of invasion. Therefore, mounting a host-defense response likely requires a broad and non-localized defense mechanism using cell surface receptors. Taken together with our quantitative results, it is likely that the increase in actin filament density is a conserved, receptor-mediated response to the perception of microbes by host plant cells.

Significantly, both bacterial and fungal MAMPs were capable of eliciting the increases in actin filament density in host plants. In this study, the flg22 peptide mimic of bacterial flagellin was sufficient to elicit a dose-dependent increase in actin filament density as early as 0–3 hpi. This fast response was not observed with a peptide mimic of the bacterial elongation factor EF-Tu (elf26), which indicates that the actin response may be specific to particular MAMPs, or could represent differential expression of MAMP receptors in organs, tissues and specific cell types [46]. For example, the EF-Tu receptor, EFR, may not be expressed in epidermal cells of cotyledons or rosette leaves but is still expressed in the whole organ. This could lead to lack of a detectable actin-based response in epidermal cells, whereas transcriptional hallmarks of PTI are still present in the whole organ. Another alternative is that the actin-based response in epidermal cells occurs rapidly and goes undetected over the timescales we are able to measure by SDC microscopy. In this way, a fast response would become “averaged-out” at the earliest timepoints measured. The increase in actin filament density still occurred in response to the *ΔfliC* mutant, indicating that additional MAMPs also trigger this response or that the presence of flagella is not completely abrogated in the *ΔfliC* mutant, despite lack of mobility. This increase in actin abundance occurs independently of the FLS2 receptor following DC3000 or *ΔfliC* treatment. Since, multiple *Pseudomonas* mutants are still capable of eliciting the increase in actin filament abundance 6–9 hpi in the absence of components of the FLS2 receptor complex, this further indicates that multiple or additional MAMPs are capable of altering host-actin architecture. Additionally, the application of fungal chitin on leaves (this study) or bacterial Nod factors on root hairs [9] also stimulated an increase in actin filament density, which further indicates that the host-actin cytoskeleton plays a general role in the perception of beneficial and pathogenic microbes.

A main hallmark of PTI is signaling through MAPK and CDPK phosphorylation cascades after host-perception of various MAMPs [4], [16]–[19]. For example, once flagellin or flg22 has bound the FLS2 receptor, BAK1 associates with FLS2, and following this association, the cytoplasmic kinase BIK1 dissociates from the receptor complex initiating host-defense signaling [16]–[19]. As expected, *Arabidopsis* mutants or ecotypes with deficiencies in the flagellin-sensing pathway (*i.e. fls2*, Ws-0) did not display changes actin filament architecture following treatment with the flg22 MAMP peptide. However, actin architecture changes still occurred following chitin treatment, indicating that chitin-induced signaling is still intact. Further, mutants in the shared signaling nodes between the flagellin- and chitin-signaling pathways (*i.e. bak1-4* and *bik1*) did not display any significant changes to actin architecture following either bacterial or fungal MAMP treatment, indicating that actin rearrangements are conserved in several common immune pathways.

### Changes in Actin Organization Are Important for Various Aspects of Plant Immunity

Our data provide evidence that the host-actin cytoskeleton plays an important role in innate immunity because the actin polymerization inhibitor LatB promotes the growth of *P. syringae* DC3000 on *Arabidopsis* leaves. Further this growth advantage is conferred specifically during PTI as the T3SS-deficient mutant *hrpH* also exhibited significantly enhanced growth. It is well known that an intact actin cytoskeleton is required for receptor-mediated endocytosis of ligands including the flagellin receptor FLS2 [47], as vesicle dynamics are reduced following treatment with either LatB or the actin stabilizer endosidin1 [48]; however, the actin cytoskeleton almost certainly plays additional roles during response to microbes. The requirement of the actin cytoskeleton for activation of NADPH oxidase at the plasma membrane, as well as Golgi, peroxisomes and endoplasmic reticulum trafficking toward sites of fungal and oomycete penetration, has been demonstrated through pharmacological studies [20]–[23]. Presumably, the trafficking of Golgi and ER is important for the production and deposition of antimicrobial compounds and fortification of the cell wall [20]–[23]. The specific targeting of defense proteins to the cell membrane is also an actin cytoskeleton-dependent process. The fungal resistance protein RPW8.2 prevents haustorium development and reduces oxidative damage to host cells by generating a unique membrane that fuses to the extrahaustorial matrix (EHM; [49]). Targeting of RPW8.2 to the membrane is disrupted with cytochalasin E treatment; plants susceptible to the powdery mildew fungus had less EHM localization and are unable to activate the same proteins as resistant plants [49]. In contrast, one actin-independent mechanism in plant defense is the accumulation of a SNARE involved in membrane fusion events at the plasma membrane, PEN1, at the fungal penetration site [20], [23], [50]. This differs from PEN2 and PEN3, which are implicated in callose deposition and require the actin cytoskeleton for proper localization during fungal infection [22], [50], [51]. Finally, perturbations to the actin cytoskeleton using drugs and toxins have been shown to trigger or alter programmed cell death in plant cells [2]. Taken together, these observations speak to the involvement of the actin cytoskeleton at various time-points, from minutes to hours, during common biotic stress events.

### The Actin Cytoskeleton—A Node Targeted by Pathogens

The regulation and turnover of the actin cytoskeleton requires the concerted activities of hundreds of actin-binding proteins that can respond to signals to polymerize or destroy actin filament networks. The growth of individual actin filaments in the cortical array of *Arabidopsis* epidermal cells is extremely fast, ∼2 µm/s, and most filaments exist for less than 30 s before being destroyed by prolific severing activity [1], [33], [52]. This constant formation and destruction of actin networks requires a huge expense of energy—on the order of millions of ATPs per second—and is thought to represent a surveillance mechanism to various biotic and abiotic stresses [1], [12]. It is easy to imagine that changes to any number of actin-binding proteins involved in actin dynamics could result in altered filament arrays; and that targeting specific aspects of the cytoskeleton would be an excellent opportunity for successful pathogen attack.

Plant actin-binding proteins respond to a plethora of second messengers in signaling cascades, including Ca^2+^, phospholipids and pH [2], [53], [54]. A potential link between the actin cytoskeleton and specific cytosolic Ca^2+^ signatures following microbial infection requires additional study [2], [55]. Further, calcium and pH fluctuations are known to occur in *Arabidopsis* during defense responses [56], [57]. Several plant actin-binding proteins have different activities as pH fluctuates from alkaline to acidic [58], [59]. Finally, there is a long history of alterations to actin filament arrays through actin-binding proteins sensing changes to concentrations and types of phospholipids like phosphatidylinositol (4,5)-bisphosphate (PIP_2_) and phosphatidic acid [60]. Phosphatidic acid is also a second messenger for plant defense responses that can activate MAPK signaling and defense genes [61]–[63] and accumulates upon treatment with various MAMPs [63]–[66].

The initiation of immunity in plants requires the concerted effort of both PTI signaling and the recognition of microbe-derived proteins evolutionarily adapted to circumvent innate immunity. A hint that the host cytoskeleton is a target for effector proteins, comes from the use of the Harpin elicitor, which triggers defense responses in host and non-host plants [67]. Specifically, Harpin elicitor treatment of grapevine cells triggers host-microtubule depolymerization within 3 hours, but has variable effects on the actin cytoskeleton [67]. The first example of a *bona fide* phytopathogenic effector protein specifically targeting the plant cytoskeleton is HopZ1a, which depolymerizes microtubules thereby disrupting the plant secretory pathway and suppressing cell wall-mediated defenses [27]. The involvement of microtubule rearrangements during PTI that results from recognition of DC3000 is unclear since this particular pathovar of *P. syringae* does not elicit changes in microtubule organization [27] and lacks *HopZ1*. Additionally, which cytoskeleton is targeted first remains an unanswered question, as DC3000 expressing HopZ1a did not disrupt the actin cytoskeleton at 16 hpi [27] and our data show changes to the actin cytoskeleton as early as 15 min after inoculation. Effector proteins likely target the actin cytoskeleton, as inoculations with the T3SS-deficient *hrpH* did not elicit the increased bundling that was obvious with DC3000 treatment. A role for actin in ETI is indicated by data from *adf4* knock-out *Arabidopsis* plants, which are unable to elicit a hypersensitive response and are susceptible to *P. syringae* expressing AvrPphB [30]. Although the exact mechanism by which ADF4 mediates resistance to bacteria carrying AvrPphB is still unknown, it has been demonstrated recently that ADF4 is required for activation of resistance to DC3000 expressing AvrPphB through control of expression of the R-gene *RPS5* [29]. Furthermore, this work correlated changes in the localization of ADF4 with the reduced expression of *FRK1* and MAPK signaling, further implying a dual role for the actin cytoskeleton in the host response to phytopathogens.

In summary, we monitored the nature and timing of changes to the actin cytoskeleton in *Arabidopsis* during microbial infection. We quantified two distinct actin responses—a rapid transient increase in actin filament density and a late increase in filament bundling. We demonstrate that the early transient increase in actin filament density is associated with PTI by using adapted and non-adapted microbes and treatments with MAMPs. We also established the requirement of host-cell signaling machinery including the flagellin receptor complex, *FLS2*, *BAK1* and *BIK1*, for the increase in actin filament abundance. This is the first evidence for temporal changes in actin cytoskeleton organization during PTI elicited by a phytopathogenic bacterium, and uncovers the initial MAMP signaling cascade responsible for altering the cytoskeleton.

## Materials and Methods

### Plant Material

*Arabidopsis thaliana* Ws-0, *fls2* (SALK_062054), *bak1-4* (SALK_116202), *bik1* (SALK_005291) were transformed with GFP-fABD2 [68] using the floral dip method described previously [69]. T1 plants were screened on appropriate antibiotics and by fluorescence microscopy. Multiple T2 plants (*n*≥9) for each genotype were used for actin architecture analysis. Wild-type *A. thaliana* Col-0, Col-0 expressing GFP-fABD2 [1], and T2 plants expressing GFP-fABD2 were sown onto soil and stratified for 3 d at 4°C. Flats were transferred to a growth chamber and plants grown under long-day conditions (16 h light, 8 h dark) at 21°C for 10 or 24 d.

### Plant Inoculations

Information about the *Pseudomonas* mutants and strains, as well as *Agrobacterium* and *Magnaporthe* strains, used in this study are found in Table S1 in Text S1. Various bacterial strains were grown on NYGA media (0.5% [w/v] Bacto Peptone, 0.3% [w/v] yeast extract, 2% [v/v] glycerol, 15% [w/v] Bacto agar) and diluted with 10 mM MgCl_2_ to a concentration of 3×10^7^ colony-forming units (CFU) mL^−1^ [70]. Ten day-old *Arabidopsis* seedlings were infected by gently agitating inverted seedlings in bacterial suspensions supplemented with of 0.02% [v/v] Silwet. MAMP peptides, flg22 [41], elf26 [43], and flg^At^ [41] all from NeoBioSci (Cambridge, MA), and chitin (Sigma-Aldrich, St. Louis, MO) were diluted in 10 mM MgCl_2_ at various concentrations. For hand infiltration of peptides, and latrunculin B co-infiltration with *Pseudomonas* strains, leaves of similar size from 24 d-old plants were designated for analysis with a marker. Leaves were gently infiltrated with an inoculum of 1×10^5^ CFU/mL using a 3 mL needle-less syringe until intercellular spaces were filled with solution (∼300–400 µL per leaf). After infiltration, inoculated plants were covered for 30 min and returned to the growth chamber prior to imaging.

### Image Acquisition and Actin Array Architecture Analysis

All image collection and data analyses were performed as single-blind experiments. Actin filament bundling and percent occupancy were measured using two metrics: *skewness*, based on the assumption that a population of actin filaments exhibits enhanced pixel intensities when bundled; and, *density*, an estimation calculated as the percent occupancy of signal (actin filaments) separated from background by setting a minimal threshold to include all actin filaments [31]. We imaged fields of epidermal pavement cells with spinning disk confocal microscopy (SDCM) by collecting 24 steps of 0.5 µm each starting at the plasma membrane. Spinning disk confocal microscopy was performed using a Yokogawa CSU-10 mounted on a Zeiss Observer Z.1 equipped with a 100X/1.46 NA PlanApo objective. Illumination was from a solid-state 50-mW laser with AOTF control over excitation wavelength (Intelligent Imaging Innovations, Denver, CO). The 488-nm laser emission was captured with an Evolve512 EMCCD camera (Photometrics, Tucson, AZ). The SDCM was operated using SlideBook software (version 5.0.031; Intelligent Imaging Innovations). A fixed specimen exposure time and gain setting were selected such that individual actin filaments could be observed, but actin filament bundles were not saturated. Maximum-intensity projections of z-series stacks were analyzed in ImageJ using algorithms described previously [31]–[33]. Gaussian blur and high-band pass filters were applied to projections prior to *density* analysis. No image processing was applied to maximum-intensity projections that were analyzed for *skewness*. At least 90 images were analyzed per time-point per treatment, from at least 30 individual seedlings for each measurement. Statistical comparisons and ANOVA test with Tukey HSD post-hoc analysis were carried out using KaleidaGraph (version 4.1.3b1; Synergy Software, Reading, PA).

## Supporting Information

Figure S1 ***Arabidopsis*** **seedlings support the growth of** ***Pseudomonas syringae*** **pv.** ***tomato*** **DC3000.** Disease phenotypes of 10–14 d-old *A. thaliana* Col-0 (**A** & **B**) and Col-0 expressing GFP-fABD2 (**C** & **D**) seedlings dip-inoculated with *P. syringae* DC3000 (**A** & **C**) and *hrpH* (**B** & **D**) are shown at 4 d-post infection (dpi). Bacterial growth was measured 0 and 4 dpi on mature rosette leaves (**E**) and seedling cotyledons (**F**) from Col-0 and GFP-fABD2 plants infected with 3×10^7^ colony-forming units (CFU) mL^−1^ of DC3000 and *hrpH*. Values given are means ± SD from 3 technical replicates. Experiments were repeated twice.
(TIF)

Figure S2 **Actin filament abundance increases rapidly in response to** ***P. syringae*** **strains.** Actin architecture in epidermal pavement cells changes rapidly in response to treatment with DC3000 and *hrpH*. DC3000- and *hrpH*-treated epidermal cells from cotyledons displayed significant increases to actin filament density (**A**) but no change to filament bundling (**B**) compared with mock control. Images were collected at 15–30 min following inoculation as described for Figure 1. Values given are means ± SE (*n* = 150 images per treatment, from *n* = 15 biological repeats). Asterisks represent significant differences by ANOVA (* = *P*≤0.05; nd = no significant difference).
(TIF)

Figure S3 **Actin architecture does not differ between mock-treated and untreated cotyledons.** Mock-treated epidermal cells from cotyledons had no significant changes to actin filament density (**A**) or filament bundling (**B**) compared to untreated epidermal cells. Images were collected at 0–3 hpi as described for Figure 1. Values given are means ± SE (*n* = 150 images per treatment, from *n* = 3 biological repeats). Asterisks represent significant differences by ANOVA (nd = no significant difference).
(TIF)

Figure S4 **Actin filament organization changes following inoculation with** ***P. syringae*** **DC3000 expressing AvrPphB.** Actin architecture parameters for percent occupancy (**A**) and extent of filament bundling (**B**) were measured in epidermal cells from cotyledons in response to inoculation with DC3000 expressing AvrPphB. Actin filament abundance in epidermal cells following AvrPphB treatment is significantly elevated compared to mock controls at each timepoint measured (**A**). Further, AvrPphB-treated seedlings have significantly elevated percent occupancy compared to DC3000 from 18 hpi onwards (**A**). The presence of actin filament bundles in epidermal cells following AvrPphB treatment is significantly elevated compared to mock treatment; however, bundling is significantly less than seedlings treated with DC3000 (**B**). Values given are means ± SE (*n* = 150 images per treatment, per timepoint, from *n* = 3 biological repeats). Significant differences by ANOVA, with Tukey HSD post-hoc analysis, are represented as follows: a, *P*≤0.05 between mock and treatment; b, *P*≤0.05 between DC3000 and treatment.
(TIF)

Figure S5 **Actin architecture changes in response to flg22 peptide treatments are dose-dependent.** Actin architecture in epidermal pavement cells exhibits a range of changes in response to treatment with various concentrations of MAMP peptides. Concentrations greater than 1 µM flg22 peptide elicited dose-dependent increases in percent occupancy compared to 0 µM treatment (**A**); however, bundling is unaltered with flg22 treatment (**B**). The elf26 (**C**) or flg^At^ (**E**) peptides did not elicit changes to percent occupancy for any concentration tested. There is also no significant change in bundling with any concentration of elf26 (**D**) or flg^At^ (**F**). Treatment with chitin oligomers elicited dose-dependent increases in filament density (**G**), whereas bundling was unchanged for any concentration tested (**H**). Images were collected as described for Figure 5. Values given are means ± SE (*n* = 150 images per treatment, from *n* = 3 biological repeats). Asterisks represent significant differences by ANOVA (nd = no significant difference; * = *P*≤0.01; ** = *P*≤0.001).
(TIF)

Figure S6 **Induction of** ***FRK1*** **expression following MAMP-peptide treatments.** Real-time quantitative PCR (RT-qPCR) was used to determine *FRK1* transcript levels in 24 d-old plants infiltrated with 1 µM flg22, elf26, or flg^At^ peptides relative to mock treatment. Treatment with flg22 or elf26 elicited a significant increase in *FRK1* transcripts compared to mock treatment or flg^At^ treatments. RT-qPCR transcripts were normalized to the housekeeping gene glyceraldehyde-3-phosphate dehydrogenase (*GAPD*). Transcript amplification of either *FRK1* or *GAPD* was absent from controls lacking reverse-transcriptase. Values given as means ± SE (*n* = 9 leaves sampled per treatment, from *n* = 3 biological and technical replicates). Asterisks represent significant differences by ANOVA, with Tukey HSD post-hoc analysis (* = *P*≤0.05; *** = *P*≤0.0001).
(TIF)

Figure S7 **Pathogenic and non-pathogenic** ***Pseudomonas*** **strains elicit an increase in actin filament abundance on** ***Arabidopsis*** **defense signaling mutants.** Actin architecture analysis of epidermal cells was performed on 10 d-old *Arabidopsis* seedlings following treatment with pathogenic and non-pathogenic *P. syringae* strains. Each *P. syringae* strain significantly elevated actin filament abundance in wild-type Col-0 plants compared to mock-treatment (**A**). Each *P. syringae* treatment also significantly elevated actin filament abundance in the *fls2* mutant (**C**) and in the Ws-0 ecotype (**E**), albeit to a lesser extent than in wild-type Col-0 plants. There was no significant change in the extent of filament bundling following treatment with any *P. syringae* strain in wild-type Col-0, the *fls2* knockout mutant, or the Ws-0 ecotype seedlings (**B, D & F**).
(TIF)

Text S1 **This file contains: Supplemental Methods; Supplemental References; Supplemental Figure Legends for Figures S1, S2, S3, S4, S5, S6, S7; and Supplemental Table S1, Microbial Strain and Mutant Description and Sources.**
(DOCX)
