# Supplementary figures and images for: The Plant Actin Cytoskeleton Responds to Signals from Microbe-Associated Molecular Patterns
Source: PLoS Pathog. 2013 Apr 4;9(4):e1003290. doi: 10.1371/journal.ppat.1003290 (PMC3616984; doi:10.1371/journal.ppat.1003290)

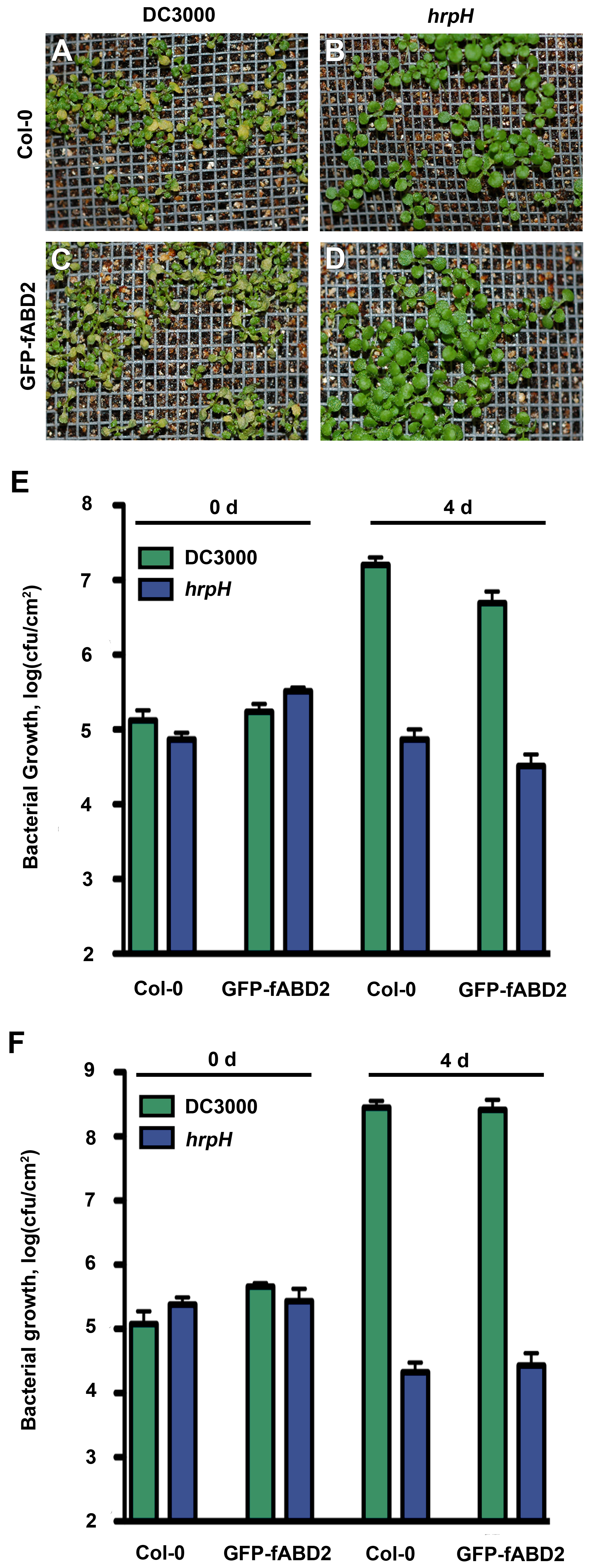

Supplement: Figure S1 — Arabidopsis seedlings support the growth of Pseudomonas syringae pv. tomato DC3000. Disease phenotypes of 10–14 d-old A. thaliana Col-0 (A & B) and Col-0 expressing GFP-fABD2 (C & D) seedlings dip-inoculated with P. syringae DC3000 (A & C) and hrpH (B & D) are shown at 4 d-post infection (dpi). Bacterial growth was measured 0 and 4 dpi on mature rosette leaves (E) and seedling cotyledons (F) from Col-0 and GFP-fABD2 plants infected with 3×10^7^ colony-forming units (CFU) mL−1 of DC3000 and hrpH. Values given are means ± SD from 3 technical replicates. Experiments were repeated twice. (TIF) [file ppat.1003290.s001.tif]

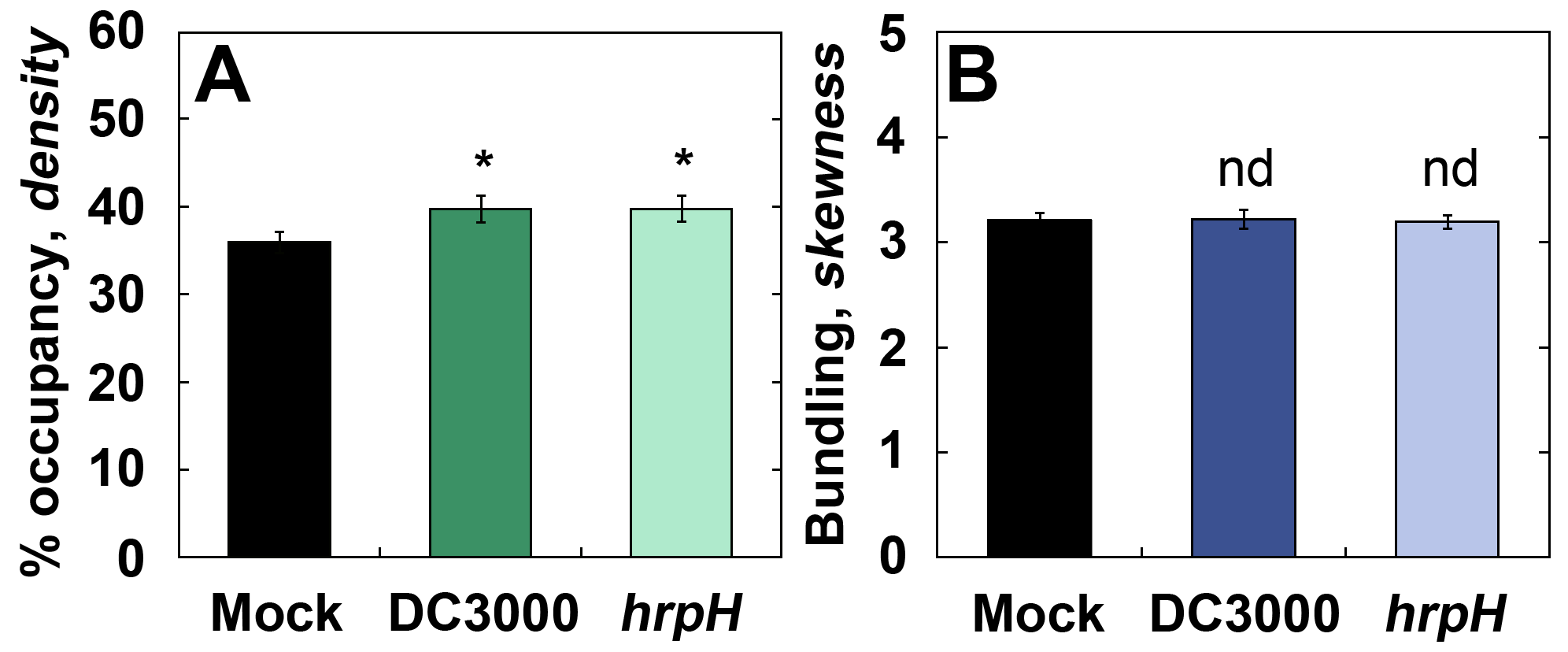

Supplement: Figure S2 — Actin filament abundance increases rapidly in response to P. syringae strains. Actin architecture in epidermal pavement cells changes rapidly in response to treatment with DC3000 and hrpH. DC3000- and hrpH-treated epidermal cells from cotyledons displayed significant increases to actin filament density (A) but no change to filament bundling (B) compared with mock control. Images were collected at 15–30 min following inoculation as described for Figure 1. Values given are means ± SE (n = 150 images per treatment, from n = 15 biological repeats). Asterisks represent significant differences by ANOVA (* = P≤0.05; nd = no significant difference). (TIF) [file ppat.1003290.s002.tif]

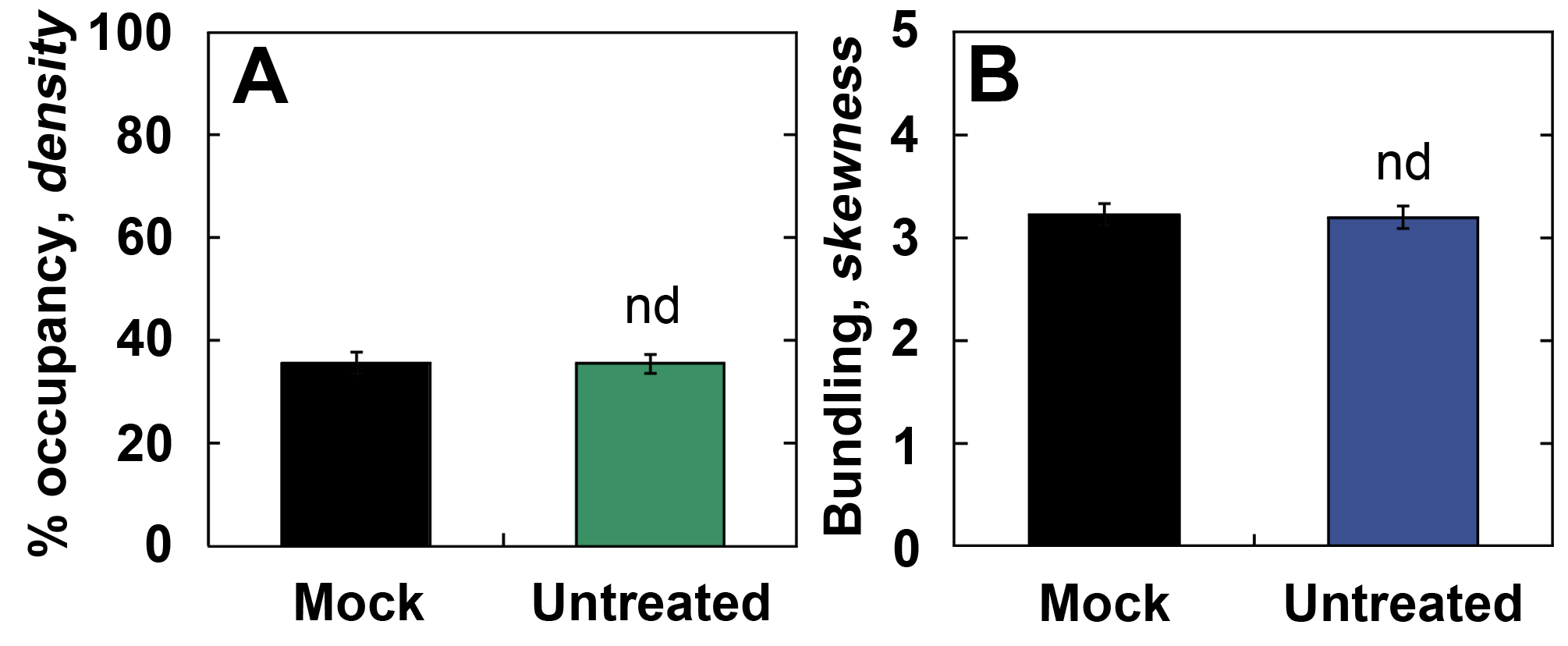

Supplement: Figure S3 — Actin architecture does not differ between mock-treated and untreated cotyledons. Mock-treated epidermal cells from cotyledons had no significant changes to actin filament density (A) or filament bundling (B) compared to untreated epidermal cells. Images were collected at 0–3 hpi as described for Figure 1. Values given are means ± SE (n = 150 images per treatment, from n = 3 biological repeats). Asterisks represent significant differences by ANOVA (nd = no significant difference). (TIF) [file ppat.1003290.s003.tif]

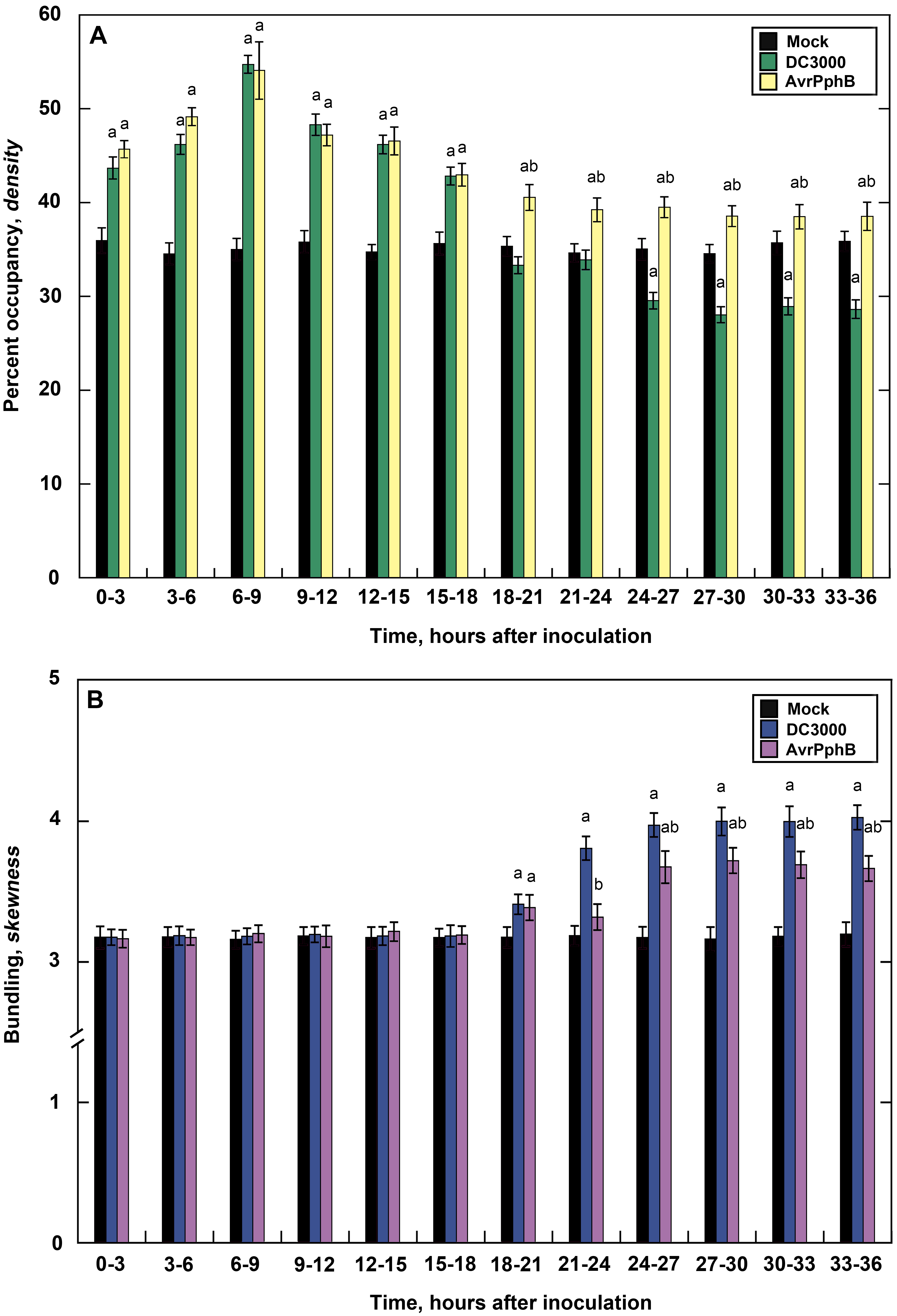

Supplement: Figure S4 — Actin filament organization changes following inoculation with P. syringae DC3000 expressing AvrPphB. Actin architecture parameters for percent occupancy (A) and extent of filament bundling (B) were measured in epidermal cells from cotyledons in response to inoculation with DC3000 expressing AvrPphB. Actin filament abundance in epidermal cells following AvrPphB treatment is significantly elevated compared to mock controls at each timepoint measured (A). Further, AvrPphB-treated seedlings have significantly elevated percent occupancy compared to DC3000 from 18 hpi onwards (A). The presence of actin filament bundles in epidermal cells following AvrPphB treatment is significantly elevated compared to mock treatment; however, bundling is significantly less than seedlings treated with DC3000 (B). Values given are means ± SE (n = 150 images per treatment, per timepoint, from n = 3 biological repeats). Significant differences by ANOVA, with Tukey HSD post-hoc analysis, are represented as follows: a, P≤0.05 between mock and treatment; b, P≤0.05 between DC3000 and treatment. (TIF) [file ppat.1003290.s004.tif]

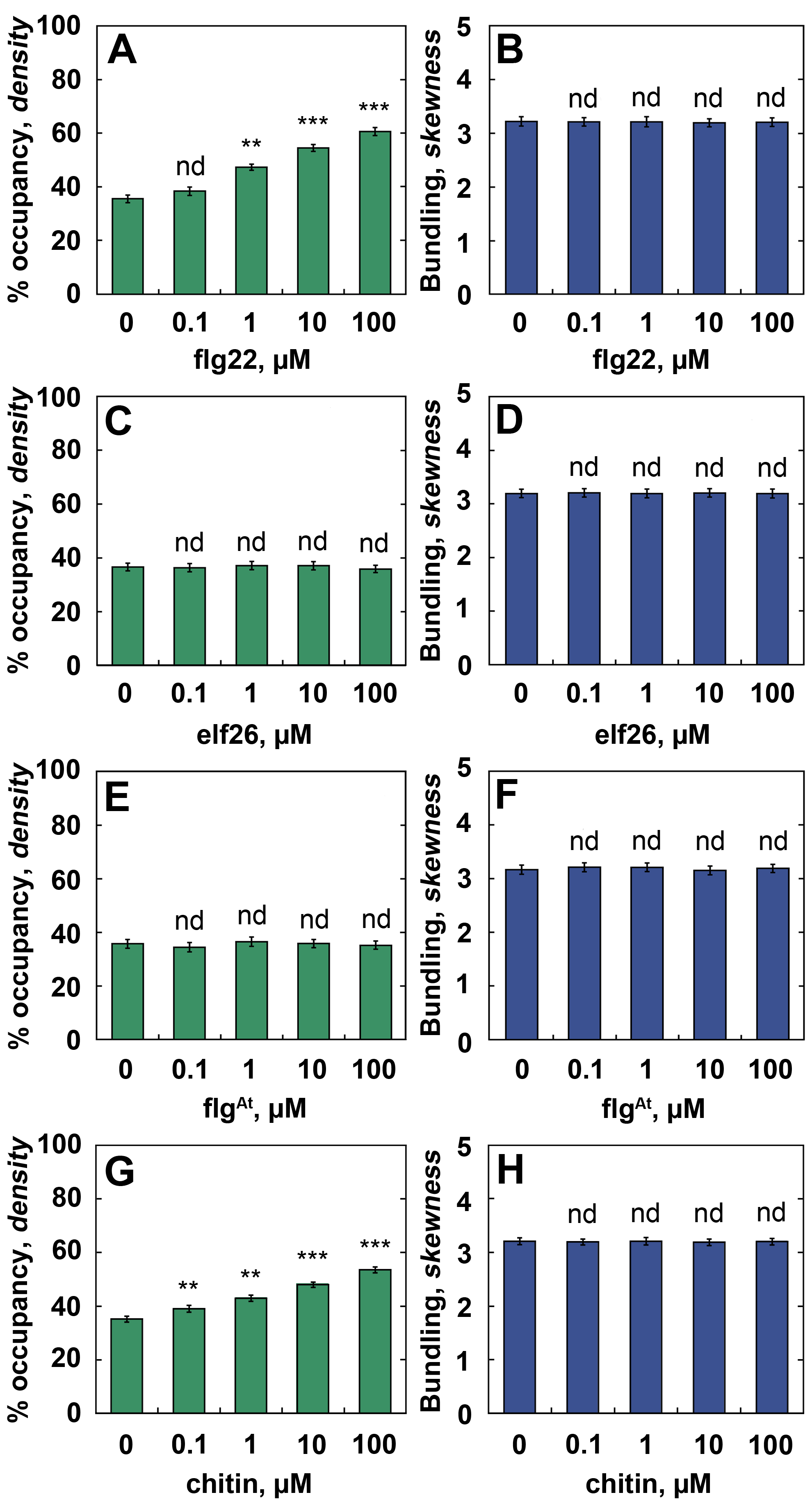

Supplement: Figure S5 — Actin architecture changes in response to flg22 peptide treatments are dose-dependent. Actin architecture in epidermal pavement cells exhibits a range of changes in response to treatment with various concentrations of MAMP peptides. Concentrations greater than 1 µM flg22 peptide elicited dose-dependent increases in percent occupancy compared to 0 µM treatment (A); however, bundling is unaltered with flg22 treatment (B). The elf26 (C) or flgAt (E) peptides did not elicit changes to percent occupancy for any concentration tested. There is also no significant change in bundling with any concentration of elf26 (D) or flgAt (F). Treatment with chitin oligomers elicited dose-dependent increases in filament density (G), whereas bundling was unchanged for any concentration tested (H). Images were collected as described for Figure 5. Values given are means ± SE (n = 150 images per treatment, from n = 3 biological repeats). Asterisks represent significant differences by ANOVA (nd = no significant difference; * = P≤0.01; ** = P≤0.001). (TIF) [file ppat.1003290.s005.tif]

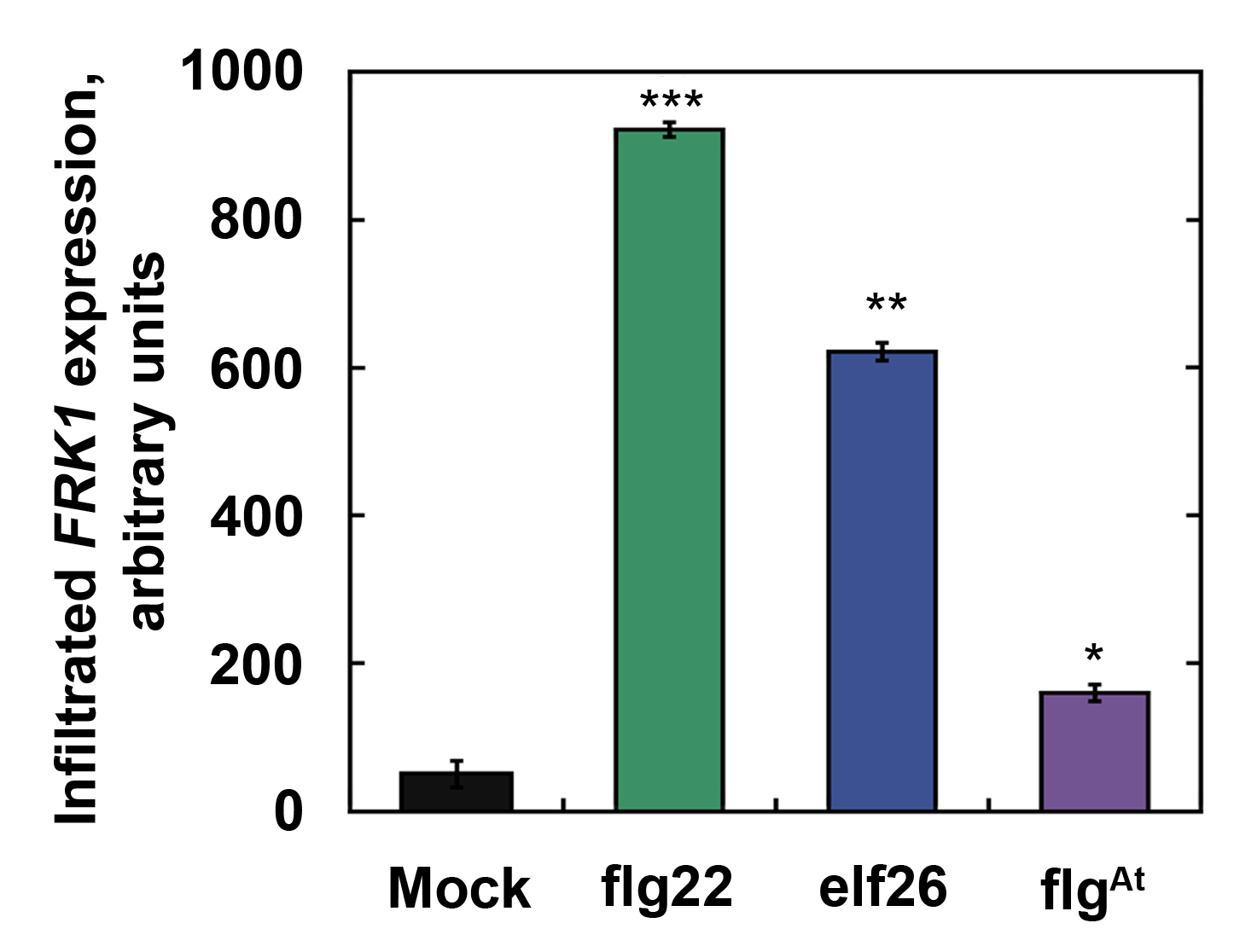

Supplement: Figure S6 — Induction of FRK1 expression following MAMP-peptide treatments. Real-time quantitative PCR (RT-qPCR) was used to determine FRK1 transcript levels in 24 d-old plants infiltrated with 1 µM flg22, elf26, or flgAt peptides relative to mock treatment. Treatment with flg22 or elf26 elicited a significant increase in FRK1 transcripts compared to mock treatment or flgAt treatments. RT-qPCR transcripts were normalized to the housekeeping gene glyceraldehyde-3-phosphate dehydrogenase (GAPD). Transcript amplification of either FRK1 or GAPD was absent from controls lacking reverse-transcriptase. Values given as means ± SE (n = 9 leaves sampled per treatment, from n = 3 biological and technical replicates). Asterisks represent significant differences by ANOVA, with Tukey HSD post-hoc analysis (* = P≤0.05; *** = P≤0.0001). (TIF) [file ppat.1003290.s006.tif]

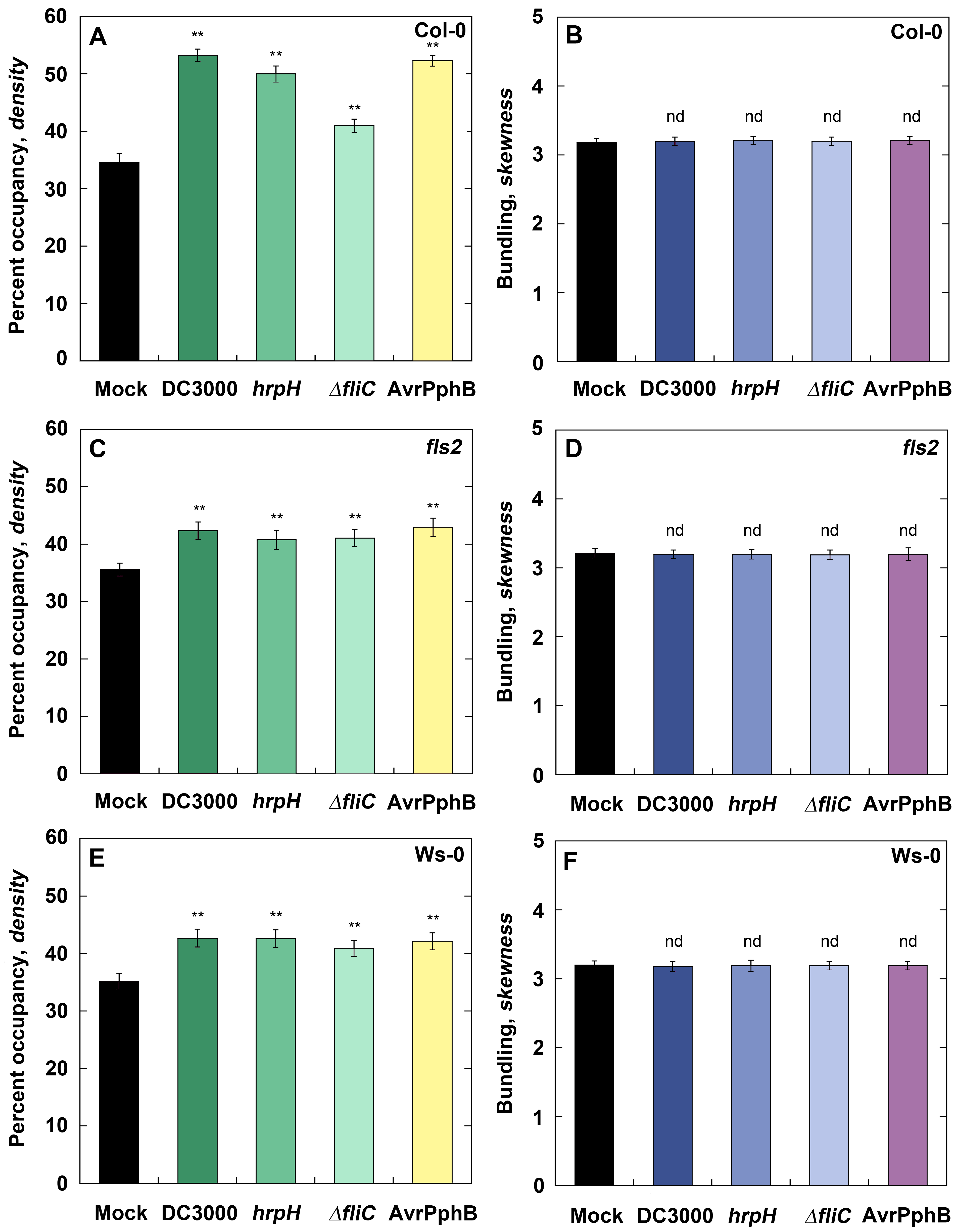

Supplement: Figure S7 — Pathogenic and non-pathogenic Pseudomonas strains elicit an increase in actin filament abundance on Arabidopsis defense signaling mutants. Actin architecture analysis of epidermal cells was performed on 10 d-old Arabidopsis seedlings following treatment with pathogenic and non-pathogenic P. syringae strains. Each P. syringae strain significantly elevated actin filament abundance in wild-type Col-0 plants compared to mock-treatment (A). Each P. syringae treatment also significantly elevated actin filament abundance in the fls2 mutant (C) and in the Ws-0 ecotype (E), albeit to a lesser extent than in wild-type Col-0 plants. There was no significant change in the extent of filament bundling following treatment with any P. syringae strain in wild-type Col-0, the fls2 knockout mutant, or the Ws-0 ecotype seedlings (B, D & F). (TIF) [file ppat.1003290.s007.tif]
