# Supplementary material for: The Plant Actin Cytoskeleton Responds to Signals from Microbe-Associated Molecular Patterns
Source: PLoS Pathog. 2013 Apr 4;9(4):e1003290. doi: 10.1371/journal.ppat.1003290 (PMC3616984; doi:10.1371/journal.ppat.1003290)
Supplement: Text S1 — This file contains: Supplemental Methods; Supplemental References; Supplemental Figure Legends for Figures S1, S2, S3, S4, S5, S6, S7; and Supplemental Table S1, Microbial Strain and Mutant Description and Sources. (DOCX) [file ppat.1003290.s008.docx]

Supporting Information

**Supplemental Methods**

**Measurement of Bacterial Growth**

*Pseudomonas syringae* pv. *tomato* DC3000 strains were grown as previously described [[1](#_ENREF_1)]. Seedlings (10­–14 d old) and five-week-old plants were inoculated in bacterial suspensions of 3 × 10^7^ colony-forming units (CFU) mL^-1^ [[2](#_ENREF_2)]. Leaf samples were collected at 0 and 4 d post-inoculation, as follows: for seedlings, 12 seedlings were harvested which represents approximately the same leaf-area of one punch (0.385 cm^2^); for five-week-old plants, 3 punches (1.155 cm^2^) were collected from a single plant. Bacterial growth assays were performed as previously described [[3](#_ENREF_3)] with modifications as explained in [[1](#_ENREF_1)].

**RNA Extraction and Real-time Quantitative PCR (RT-qPCR)**

Twenty-four d-old light-grown seedlings that were hand-infiltrated with MAMP peptides were flash-frozen and ground to a fine powder in liquid nitrogen. RNA isolation was performed with TRIzol reagent (Invitrogen) in accordance with manufacturer’s instructions. Two-step RT-qPCR was performed using 2X SYBR Green master mix (Applied Biosystems, Carlsbad, CA), normalized to *GAPD* transcript levels, and analyzed with Excel software, as described in [[4](#_ENREF_4)]. Gene-specific primers (forward primer: 5’-GGGTCAGATTTCAACAGTTGTC-3’) and (reverse primer: 5’-AATAGCAGGTTGGCCTGTAATC-3’) for *FRK1* were used to measure PTI-induced transcript levels. Three biological and technical replicates were performed per gene-specific primer set.

**Supplemental References**

1. Tian M, Chaudhry F, Ruzicka DR, Meagher RB, Staiger CJ, et al. (2009) Arabidopsis actin-depolymerizing factor AtADF4 mediates defense signal transduction triggered by the *Pseudomonas syringae* effector AvrPphB. Plant Physiol 150: 815-824.

2. Kunkel BN, Bent AF, Dahlbeck D, Innes RW, Staskawicz BJ (1993) *RPS2*, an Arabidopsis disease resistance locus specifying recognition of *Pseudomonas syringae* strains expressing the avirulence gene *avrRpt2*. Plant Cell 5: 865-875.

3. Tornero P, Dangl JL (2001) A high-throughput method for quantifying growth of phytopathogenic bacteria in *Arabidopsis thaliana*. Plant J 28: 475-481.

4. Khurana P, Henty JL, Huang S, Staiger AM, Blanchoin L, et al. (2010) *Arabidopsis* VILLIN1 and VILLIN3 have overlapping and distinct activities in actin bundle formation and turnover. Plant Cell 22: 2727-2748.

5. Cuppels DA (1986) Generation and characterization of Tn5 insertion mutations in *Pseudomonas syringae* pv. *tomato*. Appl Environ Microbiol 51: 4.

6. Lindgren RCP, Peet RC, Panopoulos N (1986) Gene cluster of *Pseudomonas syringae* pv. "*phaseolicola*" controls pathogenicity of bean plants and hypersensitivity on nonhost plants. J Bacteriol 168: 10.

7. Koncz JS (1986) The promoter of TL-DNA gene 5 controls the tissue-specific expression of chimaeric genes carried by a novel type of *Agrobacterium* binary vector. Molecular and General Genetics MGG 204: 13.

8. Liu W, Zhou X, Li G, Li L, Kong L, et al. (2011) Multiple plant surface signals are sensed by different mechanisms in the rice blast fungus for appressorium formation. PLoS Pathog 7: e1001261.

9. Yuan J, He SY (1996) The *Pseudomonas syringae* Hrp regulation and secretion system controls the production and secretion of multiple extracellular proteins. J Bacteriol 178: 6399-6402.

10. Hauck P, Thilmony R, He SY (2003) A *Pseudomonas syringae* type III effector suppresses cell wall-based extracellular defense in susceptible *Arabidopsis* plants. Proc Natl Acad Sci 100: 8577-8582.

11. Cunnac S, Chakravarthy S, Kvitko BH, Russell AB, Martin GB, et al. (2011) Genetic disassembly and combinatorial reassembly identify a minimal functional repertoire of type III effectors in *Pseudomonas syringae*. Proc Natl Acad Sci 108: 2975-2980.

12. Kvitko BH, Park DH, Velasquez AC, Wei CF, Russell AB, et al. (2009) Deletions in the repertoire of *Pseudomonas syringae* pv. *tomato* DC3000 type III secretion effector genes reveal functional overlap among effectors. PLoS Pathog 5: e1000388

13. Shao F, Merritt PM, Bao Z, Innes RW, Dixon JE (2002) A *Yersinia* effector and a *Pseudomonas* avirulence protein define a family of cysteine proteases functioning in bacterial pathogenesis. Cell 109: 575-588.

**Supplemental Figure Legends**

**Figure S1. *Arabidopsis* Seedlings Support the Growth of *Pseudomonas syringae* pv. *tomato* DC3000.**

Disease phenotypes of 10–14 d-old *A. thaliana* Col-0 (**A** & **B**) and Col-0 expressing GFP-fABD2 (**C** & **D**) seedlings dip-inoculated with *P. syringae* DC3000 (**A** & **C**) and *hrpH* (**B** & **D**) are shown at 4 d-post infection (dpi). Bacterial growth was measured 0 and 4 dpi on mature rosette leaves (**E**) and seedling cotyledons (**F**) from Col-0 and GFP-fABD2 plants infected with 3 x 10^7^ colony-forming units (CFU) mL^-1^ of DC3000 and *hrpH*. Values given are means ± SD from 3 technical replicates. Experiments were repeated twice.

**Figure S2.** **Actin Filament Abundance Increases Rapidly in Response to *P. syringae* Strains.**

Actin architecture in epidermal pavement cells changes rapidly in response to treatment with DC3000 and *hrpH*. DC3000- and *hrpH*-treated epidermal cells from cotyledons displayed significant increases to actin filament density (**A**) but no change to filament bundling (**B**) compared with mock control. Images were collected at 15–30 min following inoculation as described for Figure 1. Values given are means ± SE (*n* = 150 images per treatment, from *n* = 15 biological repeats). Asterisks represent significant differences by ANOVA (* = *P* ≤ 0.05; nd = no significant difference).

**Figure S3.** **Actin Architecture Does Not Differ Between Mock-treated and Untreated Cotyledons.**

Mock-treated epidermal cells from cotyledons had no significant changes to actin filament density (**A**) or filament bundling (**B**) compared to untreated epidermal cells. Images were collected at 0–3 hpi as described for Figure 1. Values given are means ± SE (*n* = 150 images per treatment, from *n* = 3 biological repeats). Asterisks represent significant differences by ANOVA (nd = no significant difference).

**Figure S4. Actin Filament Organization Changes Following Inoculation with *P. syringae* DC3000 expressing AvrPphB.**

Actin architecture parameters for percent occupancy (**A**) and extent of filament bundling (**B**) were measured in epidermal cells from cotyledons in response to inoculation with DC3000 expressing AvrPphB. Actin filament abundance in epidermal cells following AvrPphB treatment is significantly elevated compared to mock controls at each timepoint measured (**A**). Further, AvrPphB-treated seedlings have significantly elevated percent occupancy compared to DC3000 from 18 hpi onwards (**A**). The presence of actin filament bundles in epidermal cells following AvrPphB treatment is significantly elevated compared to mock treatment; however, bundling is significantly less than seedlings treated with DC3000 (**B**). Values given are means ± SE (*n* = 150 images per treatment, per timepoint, from *n* = 3 biological repeats). Significant differences by ANOVA, with Tukey HSD post-hoc analysis, are represented as follows: nd = no significant difference from mock; a, *P* ≤ 0.05 between mock and DC3000; b, *P* ≤ 0.05 between mock and AvrPphB; c, *P* ≤ 0.05 between DC3000 and AvrPphB.

**Figure S5. Actin Architecture Changes in Response to flg22 Peptide Treatments are Dose-dependent.**

Actin architecture in epidermal pavement cells exhibits a range of changes in response to treatment with various concentrations of MAMP peptides. Concentrations greater than 1 µM flg22 peptide elicited dose-dependent increases in percent occupancy compared to 0 µM treatment (**A**); however, bundling is unaltered with flg22 treatment (**B**). The elf26 (**C**) or flg^At^ (**E**) peptides did not elicit changes to percent occupancy for any concentration tested. There is also no significant change in bundling with any concentration of elf26 (**D**) or flg^At^ (**F**). Treatment with chitin oligomers elicited dose-dependent increases in filament density (**G**), whereas bundling was unchanged for any concentration tested (**H**). Images were collected as described for Figure 5. Values given are means ± SE (*n* = 150 images per treatment, from *n* = 3 biological repeats). Asterisks represent significant differences by ANOVA (nd = no significant difference; * = *P* ≤ 0.01; ** = *P* ≤ 0.001).

**Figure S6. Induction of *FRK1* Expression Following MAMP-peptide Treatments.**

Real-time quantitative PCR (RT-qPCR) was used to determine *FRK1* transcript levels in 24 d-old plants infiltrated with 1 µM flg22, elf26, or flg^At^ peptides relative to mock treatment. Treatment with flg22 or elf26 elicited a significant increase in *FRK1* transcripts compared to mock treatment or flg^At^ treatments. RT-qPCR transcripts were normalized to the housekeeping gene glyceraldehyde-3-phosphate dehydrogenase (*GAPD*). Transcript amplification of either *FRK1* or *GAPD* was absent from controls lacking reverse-transcriptase. Values given as means ± SE (*n* = 9 leaves sampled per treatment, from *n* = 3 biological and technical replicates). Asterisks represent significant differences by ANOVA, with Tukey HSD post-hoc analysis (* = *P* ≤ 0.05; *** = *P* ≤ 0.0001).

**Figure S7. Pathogenic and Non-pathogenic *Pseudomonas* Strains Elicit an Increase in Actin Filament Abundance on *Arabidopsis* Defense Signaling Mutants.**

Actin architecture analysis of epidermal cells was performed on 10 d-old *Arabidopsis* seedlings following treatment with pathogenic and non-pathogenic *P. syringae* strains. Each *P. syringae* strain significantly elevated actin filament abundance in wild-type Col-0 plants compared to mock-treatment (**A**). Each *P. syringae* treatment also significantly elevated actin filament abundance in the *fls2* mutant (**C**) and in the Ws-0 ecotype (**E**), albeit to a lesser extent than in wild-type Col-0 plants. There was no significant change in the extent of filament bundling following treatment with any *P. syringae* strain in wild-type Col-0, the *fls2* knockout mutant, or the Ws-0 ecotype seedlings (**B, D & F**).

**Table S1. Microbial Strain and Mutant Descriptions and Sources.**

| **Strain** | **Strain background** | **Description** | **Source** |
| --- | --- | --- | --- |
| DC3000 | *Pseudomonas syringae* pv. *tomato* DC3000 (empty vector) | *A. thaliana* pathogen | [[5](#_ENREF_5)] |
| *Pph* | *P. syringae* pv. *phaseolicola* | Bean pathogen; non-adapted to *A. thaliana* | [[6](#_ENREF_6)] |
| *A. tum.* | *Agrobacterium tumefaciens GV3101* | Pathogenic gram-negative bacterium | [[7](#_ENREF_7)] |
| *M. grisea* | *Magnaporthe grisea Guy11* | Rice-blast fungus | [[8](#_ENREF_8)] |
| *hrpH* | *P. syringae* DC3000 *hrpH* | Non-pathogenic; T3SS-deficient mutant | [[9](#_ENREF_9)] |
| *hrcC* | *P. syringae* DC3000 *hrcC* | Non-pathogenic; T3SS-deficient mutant | [[10](#_ENREF_10)] |
| D28E | *P. syringae* DC3000 D28E | Non-pathogenic; 28 effector genes deleted; T3SS intact | [[11](#_ENREF_11)] |
| COR- | *P. syringae* DC3000 COR- | Deficient for coronatine | [[10](#_ENREF_10)] |
| *ΔfliC* | *P. syringae* DC3000 *ΔfliC* | Deficient for *fliC* and bacterial motility | [[12](#_ENREF_12)] |
| AvrPphB | *P. syringae* DC3000 *+* AvrPphB | Cognate effector for *RPS5*; elicits ETI on *Arabidopsis* | [13] |
